# Functional Profiling of CFTR-Directed Therapeutics Using Pediatric Patient-Derived Nasal Epithelial Cell Models

**DOI:** 10.3389/fped.2020.00536

**Published:** 2020-09-04

**Authors:** Jeffrey KiHyun Park, Anura Shrivastava, Chengkang Zhang, Brian A. Pollok, Walter E. Finkbeiner, Elizabeth R. Gibb, Ngoc P. Ly, Beate Illek

**Affiliations:** ^1^UCSF Benioff Children's Hospital Oakland, Children's Hospital Oakland Research Institute, Oakland, CA, United States; ^2^Propagenix Inc., Gaithersburg, MD, United States; ^3^Department of Pathology, UCSF and Zuckerberg San Francisco General Hospital, San Francisco, CA, United States; ^4^Department of Pediatrics, UCSF Benioff Children's Hospital San Francisco, San Francisco, CA, United States

**Keywords:** Cystic fibrosis, mutation-specific CFTR function analysis, CFTRdele2, 3(21kb) (c.54-5940_270+10250del21 kb, p.Ser18ArgfsX16), c.850dupA (977insA, p.Met284Asnfs), R334W (c.1000C>T, p.Arg334Trp), 406-1G->A (c.274-1G>A), R117H-7T (c.350G>A, p.Arg117His), F508del (c.1521_1523delCTT, p.Phe508del)

## Abstract

Functional profiling of CFTR-directed therapeutics offers the potential to provide significant benefits to young people with cystic fibrosis (CF). However, the development of 2D airway epithelial cell models for individual response tests in CF children remains a central task. The objective of this study was to determine the utility of EpiX^TM^ technology for expansion of nasal epithelial cells for use in electrophysiological CFTR function measurements. An initial harvest of as few as 20,000 cells was sufficient to expand up to 50 million cells that were used to generate air-liquid interface (ALI) cultures for ion transport studies with the Ussing assay. CFTR function was assessed by measuring responses to forskolin and the CFTR potentiator VX-770 (ivacaftor) in ALI cultures generated from passage 3 and 4 cells. Short-circuit current (Isc) measurements of blocked CFTR currents (ΔI_CFTRinh_) discriminated CFTR function between healthy control (wild type, WT) and patients with intermediate (F508del/R117H-7T: 56% WT) and severe (F508del/F508del: 12% WT) CF disease. For the mixed genotypes, CFTR activity for F508del/c.850dupA was 12% WT, R334W/406-1G>A was 24% WT, and CFTRdele2,3(21 kb)/CFTRdele2,3(21 kb) was 9% WT. The CFTR correctors VX-809 (lumacaftor) and VX-661 (tezacaftor) significantly increased CFTR currents for F508del/R117H to 73 and 67% WT, respectively. Cultures with the large deletion mutation CFTRdele2,3(21 kb) unexpectedly responded to VX-661 treatment (20% WT). Amiloride-sensitive sodium currents were robust and ranged between 20–80 μA/cm^2^ depending on the subject. In addition to characterizing the electrophysiological profile of mutant CFTR activity in cultures for five genotypes, our study exemplifies the promising paradigm of bed-to-bench side cooperation and personalized medicine.

## Introduction

Cystic Fibrosis (CF; OMIM 219700) is an autosomal recessive disorder caused by mutations in the gene coding the cystic fibrosis transmembrane conductance regulator (CFTR) which functions as a chloride and bicarbonate-permeable ion channel protein in the apical cell membranes of various epithelia, including the lungs, pancreas, and sweat glands (1, 2). Patients with CF have salty skin at birth and suffer from various early-onset symptoms resulting from defective ion conductance, such as respiratory mucus buildup, bacterial infections, and pancreatic insufficiency.

CF is a promising case for personalized medicine due to its genomic variety (3). As of January 2020, over 89,000 patients were registered worldwide for CF (http://www.cftr2.org/), and a total of 2,092 CFTR mutations were identified; of these, 352 were described as CF-causing (4). However, the majority of CFTR mutations and resulting variety of genotypes have not been experimentally evaluated in terms of their functional consequences on chloride and bicarbonate transport function. The majority of patients are eligible for a well-characterized treatment regimen developed for carriers with one or two F508del alleles, the most common CF variant (5); however, some remaining 40% of patients who are carriers of rare variants have no defined treatment (6), and may benefit from *ex vivo* patient-specific CFTR functionality testing. In this study, we report the results of a personalized procedure in which patient-derived cells are used to characterize mutant CFTR function and pharmacodynamic response. We examined the electrophysiologic properties of rare genotypes of five pediatric CF patients, whose cells were harvested by a swift nasal swab procedure and then expanded for *in vitro* analysis.

Nasal epithelial cells were collected from CF patients who were between 3 and 20 years old. The isolated nasal cells were subsequently expanded in culture via the novel EpiX^TM^ technology at Propagenix Inc., MD, which has previously been established as a viable method for providing functional cells for the assay (7). The EpiX^TM^ cell expansion method has been shown to successfully conserve physiological function in human bronchial epithelial cells, including CFTR function and modulator responsiveness after multiple passages (7).

The current paradigm of treatment for CF often entails the administration of small-molecule CFTR modulators, which include CFTR *potentiators* – such as ivacaftor (VX-770) – that bind to CFTR and improve its open channel probability, and *correctors* – such as tezacaftor (VX-661) and lumacaftor (VX-809) – that improve CFTR trafficking and localization to the apical plasma membrane. Often, a combination of CFTR corrector and potentiator compounds is prescribed, as in the case for homozygotes of the most common CF variant, F508del (5). In this study, nasal epithelial cultures were studied for their pharmacological responses to the following combination of CFTR modulators: VX-770 (ivacaftor, Kalydeco®) only, VX-770+VX-809 (ivacaftor/lumacaftor; Orkambi®), and VX-770+VX-661 (ivacaftor/tezacaftor; Symdeko®) (8), which were the CF modulators available during the time of this study.

To characterize CFTR function in EpiX^TM^-expanded nasal cells, we used the Ussing Chamber technique, an established electrophysiological assay for vectorial ion transport measurements in epithelial tissues. Short-circuit current measurements provide a sensitive read-out for the apical membrane chloride conductance and CFTR-mediated chloride current measurements have been established in cultured airway epithelial cells (9, 10) as well as cultures from nasal scrapings (11). Over the past years, functional responses to CFTR-directed therapeutics have emerged as a basis for preclinical studies using primary airway cell cultures (6, 12, 13), often expanded by conditional reprogramming technology in presence of 3T3 feeder cells (14–17) and more recently, by emerging feeder-free technologies (7, 17, 18). CFTR modulator theratyping has been introduced to group and match CFTR-directed medications to CF mutations. The concept is promising and enables the design of efficacious therapeutic intervention in CF patients (5, 19, 20).

**Figure 1:**
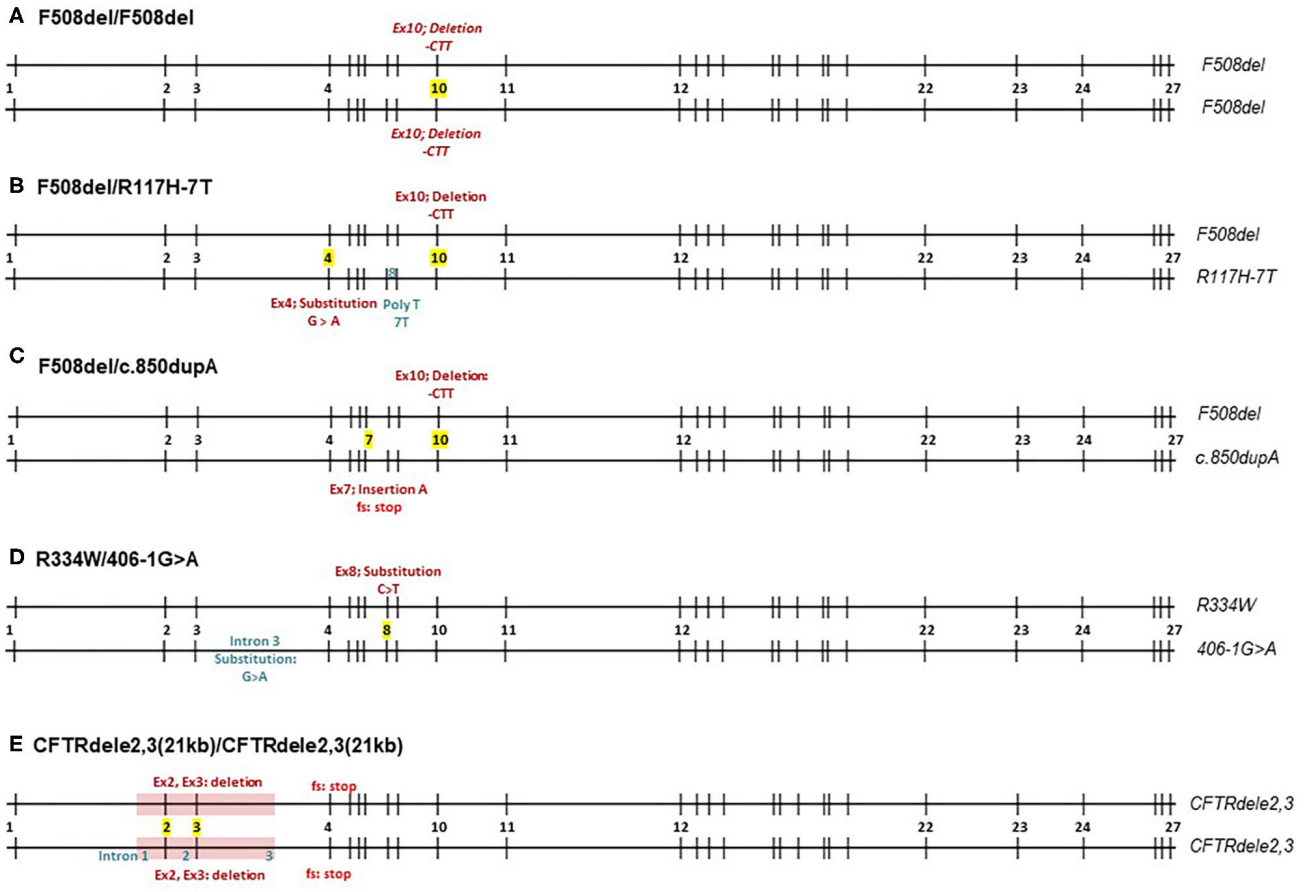
Schematic overview of CFTR mutations by five different CF genotypes. Numbers 1-27 represent exons of the CFTR gene. **(A–E)** F508del homozygote, F508del/R117H-7T, F508del/c.850dupA, R334W/406-1G>A, and CFTRdele2,3(21kb) homozygote.

In this study, we characterize the CFTR function of five different genotypes: F508del/F508del, CFTRdele2,3(21 kb)/CFTRdele2,3(21 kb), F508del/R117H-7T, R334W/406-1G>A, and F508del/c.850dupA. The variants examined in this study are briefly introduced below and the genomic structure is schematically shown in Figure 1.

*F508del* (c.1521_1523delCTT, p.Phe508del) is the deletion of a CTT sequence from nucleotide positions 1,521–1,523 in exon 10 resulting in a deletion of phenylalanine in amino acid position 508 that affects the first nucleotide binding domain of the CFTR protein (21). It represents 55% of all alleles (99,061/178,104 alleles) recorded in the CFTR2 database, which accounts for CF registries in the US and Europe (4). Interestingly, the spectrum of CFTR mutations varies among different populations, ethnic backgrounds, and geographical locations.

*CFTRdele2,3(21 kb)* (c.54-5940_270+10250del21kb, p.Ser18ArgfsX16) is an example of a less common CF mutation that is frequently observed in Central and Eastern Europe (1.1–6.4%) and in particular among Slavic populations (22). It represents <1% of all alleles (417/178,104 alleles) recorded in the CFTR2 database, which accounts for CF registries in the US and Europe (4) and is associated with severe CF symptoms (22). The mutation is a large deletion mutation of 21 kb of the region spanning exons 2 and 3 of the CFTR gene including adjacent introns 1, 2, and 3. This deletion leads to a frameshift downstream and a premature stop signal in codon 106, producing a potential product that is 32 amino acids long with no transmembrane or gating domains. The existing literature suggests that CFTRdele2,3(21 kb) produces truncated CFTR mRNA lacking exons 2 and 3, however no electrophysiological studies have been performed to examine the level of CFTR function in CFTRdele2,3(21 kb).

F508del/R117H-7T is a genotype that has been extensively studied. The R117H mutation (c.350G>A, p.Arg117His) is caused by the substitution of guanine to adenine at nucleotide 350 in exon 4, changing arginine to histidine at residue 117 which is located in the extracellular loop that connects the first and second membrane-spanning regions of the first transmembrane domain. In this study, the R117H mutation is *cis* to a 7T polymorphism in the poly T-tract of intron 8 with a reported allele frequency of 125/178,104 alleles (4). The 7T often results in less severe CF phenotype, as opposed to 3T or 5T repeats (23, 24). R117H is clinically treated with ivacaftor.

R334W/406-1G>A. *R334W* (c.1000C>T, p.Arg334Trp) is an uncommon mutation with an allelic frequency of 0.2% [429/178,104; (4)]. It causes a substitution of cytosine to thymine at nucleotide position 1,000 in exon 8. The R334W mutation is located within the sixth membrane-spanning region of the first transmembrane domain and has a high conductivity for bicarbonate (25).

*406-1G*>*A* (c.274-1G>A) is a very uncommon mutation with an allelic frequency of 0.02% [40/178,104 alleles; (4)] that has been identified in the Hispanic CF population in the US (26). It belongs to a canonical splice mutation where the guanine is changed to adenine at one nucleotide before cDNA locus 274, in intron 3. This causes an mRNA splicing defect and presumably no proper post-translational processing of CFTR (26).

F508del/c.850dupA. *c.850dupA* (977insA, p.Met284Asnfs) is an extremely uncommon mutation with an allelic frequency of 0.004% [only 8 alleles/178,104; (4)]. It causes an insertion of adenine at nucleotide position 850 in exon 7 that creates a stop codon two triplets downstream due to frameshift. The stop codon presumably leads to termination of CFTR translation at the cytosolic polypeptide chain following the 4th membrane-spanning region of the first transmembrane domain (27).

We report that the genotypes studied here range from intermediate to low CFTR functionality, with F508del/R117H-7T retaining as much as 56% wildtype CFTR function, R334W/406-1G>A retaining 24%, F508del/c.850dupA retaining 12%, F508del/F508del retaining 12%, and CFTRdele2,3(21 kb)/CFTRdele2,3(21 kb) retaining 9%. Among the modulators considered in this study, VX-770+VX-809 (Orkambi®) was most effective in rescuing F508del/R117H-7T and F508del/c.850dup, and less in F508del/F508del and R334W/406-1G>A. Unexpectedly, VX-770+VX-661 (Symdeko®) showed effects on homozygous CFTRdele2,3(21 kb). These findings extend our understanding of the five genotypes studied, some of which are rare and understudied in the standard literature, and exemplify the efficacy of personalized medicine as a paradigm for CF treatment.

## Materials and Methods

### Nasal Swab and Initial Cell Harvest

Nasal cell procurement was performed at UCSF Benioff Children's Hospital Oakland in Oakland, CA on five patients with the following genotypes: *F508del/R117H-7T* (male, 6 year old, Caucasian, not on CFTR modulators), *R334W/406-1G*>*A* (female, 3 year old, not on CFTR modulators), *F508del/c.850dupA* (female, 20 year old, Caucasian, on Symdeko), *F508del/F508del* (male, 17 year old, Caucasian, not on CFTR modulators), *CFTRdele2,3(21 kb)/CFTRdele2,3(21 kb)* (female, 4 year old, Caucasian, not on CFTR modulators). Mean age was 10 years old (SE = 3.5), 60% (3 of 5 patients) identified as female, and 80% (4 of 5) identified as Caucasian. The procedure was conducted under an IRB approved protocol. Cells were obtained by a simple nasal swab using standard cytology brushes (CYB-1, Medical Packaging Corporation, Inc., sterile). No topical anesthesia was required. Brush tips were cut and placed in 10 mL transport media (Table S1).

### Expansion by EpiX^TM^ Technology

EpiX^TM^ expansion was conducted by Propagenix Inc., as previously described (7). Cells from nasal brushing samples were resuspended by multiple rounds of centrifugation, which involved trypsinization and neutralization. Total number of viable cells was counted using a hemocytometer. Harvested cells were then seeded in Collagen I-coated culture vessels, and grown in the EpiX^TM^ medium. EpiX^TM^ media was changed every 2–3 days for 26–31 days, yielding a final cell count of 50 million cells per patient's cell line (Table 1).

**Table 1:** Clinical characteristics of CF nasal cell donors and expansion of CF nasal cells by EpiX^TM^ technology.

**Genotype**	**Age (years)**	**Sex**	**Sweat NaCl (mM)**	**FEV-1%**	**Number of brushed cells**	**Days to 50 million cells**	**Passage number**	**Population doublings**
F508del/R117H-7T	6	M	45	71	1,216,000^BC^	29	3	8.7
F508del/F508del	17	M	103	56	596,000	26	3	10.4
F508del/c.850dupA	20	F	108	109	20,000	31	4	11.6
R334W/406-1G>A	3	F	113	N/A	52,000	29	3	8.7
CFTRdele2,3/CFTRdele2,3	4	F	>100	N/A	100,000	29	3	10.4

*Cells were expanded in EpiX^TM^ media over a period of 26–31 days. Population doublings refers to the total number of times that cells in a population have doubled. Sweat test and lung function measurements (FEV1%) are listed from available patient data prior to cell collection. N/A, not available; BC, sample contained red blood cells*.

### Air Liquid Interface Differentiation and Cell Culture

Expanded cells were plated onto Snapwell Inserts and differentiated at an air-liquid interface (ALI) with the Vertex Differentiation Medium, named V-ALI (Table S2) (15) for 10–15 days. Differentiated cells were then sent to UCSF Children's Hospital Oakland Research Institute for ion transport measurements in Ussing chambers. Prior to the electrophysiological studies, some cultures were incubated overnight (16–24 h) with VX-809 or VX-661 (3–30 μM) while others remained untreated, as part of the experimental design for testing combination therapies (8).

### Using Chamber Short Circuit Assay

Snapwell inserts were mounted via a slider into modified Ussing chambers (Easy Mount Chamber Systems, Physiologic Instruments, San Diego, CA). Fluid volume was 5 ml on each side. The composition of the basolateral Ussing chamber solution was (in mM): 120 NaCl, 20 NaHCO_3_, 5 KHCO_3_, 1.2 NaH_2_PO_4_, 5.6 glucose, 2.5 CaCl_2_, and 1.2 MgCl_2_. In the mucosal solution, all Cl^−^ salts were exchanged for gluconate salts. Both hemi-chambers were gassed with 5% CO_2_ /air and bath temperature was maintained at 35–37°C.

Transepithelial voltage was clamped to zero volts using a standard four-electrode voltage clamp (Physiologic Instruments, San Diego, CA). Short-circuit current was recorded to a computer through an analog-to-digital board (DI710, DataQ Instruments, Akron, OH). At 60 s intervals, transepithelial voltage was clamped to 1 mV for 1 s to calculate transepithelial electrical resistance (TER). Higher resistance represents tighter cultures. Current deflections are shown in Ussing traces to visualize TER of each culture and large current amplitudes indicate lower TER.

After short-circuit currents had stabilized at the beginning of the experiment, each culture was treated with the following protocol: ENaC inhibitor amiloride (100 μM, to mucosa), cAMP agonist forskolin (20 μM, to serosa), CFTR potentiator VX-770 (1 μM, to mucosa), CFTR inhibitors CFTRinh-172 (50 μM, to mucosa), PPQ-102 (50 μM, to mucosa), GlyH-101 (50 μM, to mucosa) (28), and Ca-activated chloride channel (CaCC) activator ATP (500 μM, to mucosa). Approximately 5–15 min elapsed between each addition until stabilization of currents was observed.

### Data Analysis and Metrics

As a non-CF control, human bronchial epithelial (HBE) cells were obtained from a patient with idiopathic pulmonary fibrosis. A frozen aliquot of cells at passage 0 was expanded for one passage via the same EpiX^TM^ technology and protocol explained above. Cells were passaged once, then differentiated in a separate differentiation medium for 28 days, followed by an identical Ussing Chamber protocol.

The standard for 100% normal CFTR function was determined by quantifying the magnitude of the forskolin-stimulated response in non-CF control cells and taking the difference between the forskolin-stimulated current (I_Fsk_) and the final current that remained after CFTR inhibitors were added (I_CFTRinh_). This approach provides a more appropriate quantification of baseline CFTR activity because it accounts for pre-existing CFTR currents. Total CFTR function in CF cells was similarly assessed for each genotype by measuring the CFTR blocker -sensitive current, which was defined as the total change in current in response to sequential additions of CFTR inhibitors (I_CFTRinh_) after combined stimulation by forskolin plus VX-770 (I_Fsk+VX-770_). Based on these parameters, we calculated % of normal CFTR function, whereby a higher percentage represents greater rescue of CFTR toward a normal phenotype, based on the following equation:

$$\begin{array}{l} \%\;normal\;CFTR\;Function\\ \;=\left|\frac{{\text{I}}_{\text{Fsk}+\text{VX}\text{-}}770\text{-}I_{CFTRinh}\;in\;CF\;cells}{{\text{I}}_{\text{Fsk}}\text{-}I_{CFTRinh}\;in\;non\text{-}CF\;cells}\right|\times \;100 \end{array}$$

The use of HBE cells for non-CF control and HNE cells for CF cultures in determining percent WT function was considered as a reasonable approach because CFTR function of bronchial epithelial cells are in a comparable range when compared to nasal epithelial cells (29) (Figure S7).

Statistical significance of treatment effects was evaluated by unpaired *t*-tests, or as otherwise stated, using Sigmaplot, version 11.0. A *p* < 0.05 was considered significant.

## Results

### EpiX^TM^ Expansion of Patient-Derived Nasal Epithelial Cells

**Figure 2:**
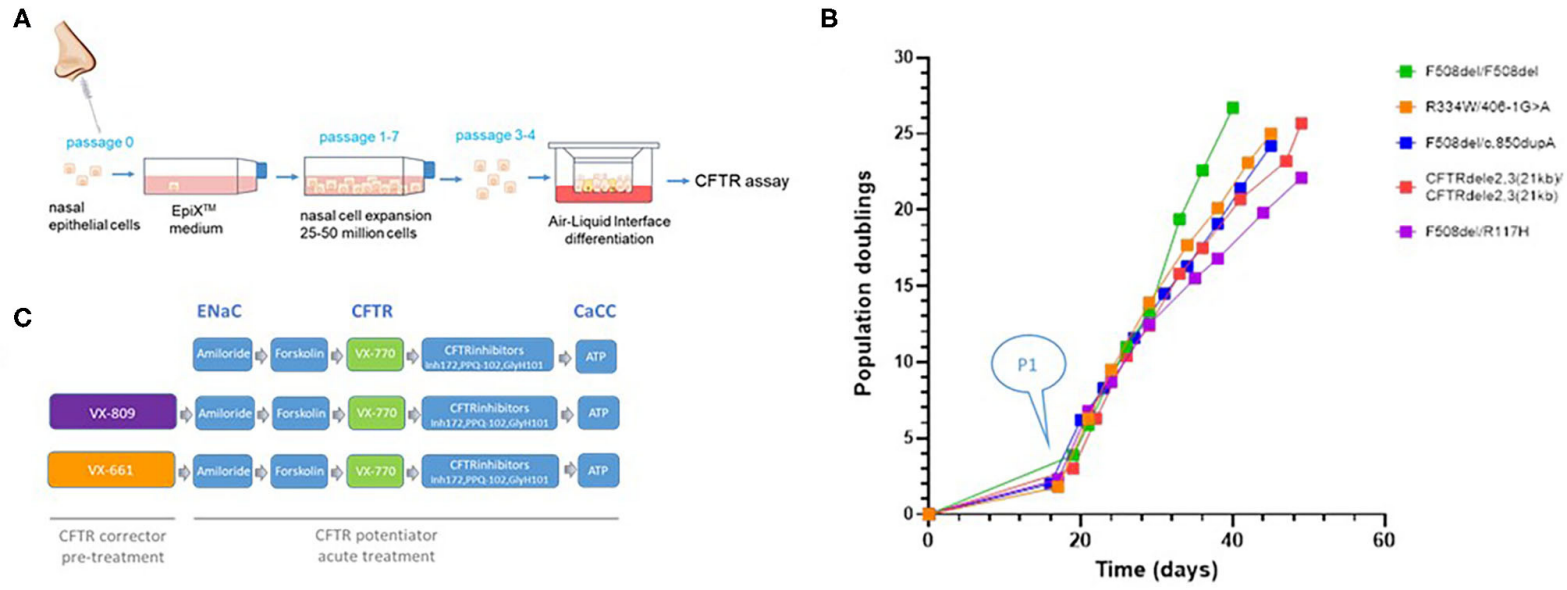
Schematic overview of nasal cell expansion in EpiX^TM^ medium and Ussing chamber protocol. **(A)** Nasal epithelial cells were collected from five CF individuals by nasal brushings. Isolated primary cells were expanded with the EpiX^TM^ method in Keratinocyte-SFM medium with TGF-β inhibitor A83-01 (1 uM), Rho-Kinase inhibitor Y-27632 (5 uM), isoproterenol (3 uM), and low calcium concentrations (CaCl_2_; 90 uM), as previously described (6). Expanded cells were plated on Snapwell inserts at Passage 3 or 4 and subsequently grown in a serum-free medium at an air-liquid interface for 21–28 days for differentiation. **(B)** Population doublings. The initial “lag phase” illustrates the number of days it took to generate CF airway cultures (passage 1) from the primary cells (P0) that were isolated from the brushed tissue sample. Each CF culture reached ~25 population doublings within 25–35 days. **(C)** Activities for ENaC, CFTR, and CaCC were assessed in non-treated cultures and cultures treated with CFTR corrector VX-809 or VX-661. CFTR activity was assessed by measuring cAMP-dependent activation by forskolin followed by acute addition of CFTR potentiator VX-770. CFTR inhibitor compounds were used to identify CFTR-mediated chloride currents. Epithelial Na channel activity was quantified by amiloride. Calcium-activated chloride currents (CaCC) were activated by ATP and measured in the presence of CFTR inhibitors.

Human nasal epithelial (HNE) cells were obtained from five CF patients by a non-invasive brushing procedure and expanded by the EpiX^TM^ technology. Initial cell counts of each individual's nasal brushing sample are listed in Table 1 and ranged from 2 × 10^4^ to 1.2 × 10^6^ cells. Figure 2A illustrates a schematic overview of the nasal cell expansion protocol. Primary nasal cells were grown to passage 1 within 2 weeks and further expanded in EpiX^TM^ medium for ~25 population doublings. Each patient's isolate reached 25 population doublings within 25–35 days (Figure 2B). Population doublings per day are shown in Figure S1 and remained stable (between 0.5 and 1.5 doublings/day) up to 7–9 passages for each isolate. Expansion of CF nasal cells with the EpiX^TM^ medium yielded ~50 million cells after 26–31 days in culture. Cells from passages 3 or 4 were grown on Snapwell inserts to generate differentiated air-liquid interface (ALI) cultures that were further analyzed in Ussing chambers. The experimental design for personalized CFTR function measurements in CF patient cultures is shown in Figure 2C. Amiloride-sensitive sodium channel (ENaC) and calcium-activated chloride channel (CaCC) activities were included in the protocol and provided as Supplementary Material (Figures S2–S6).

### Preliminary Screening of Inserts With Low Transepithelial Resistance

**Figure 3:**
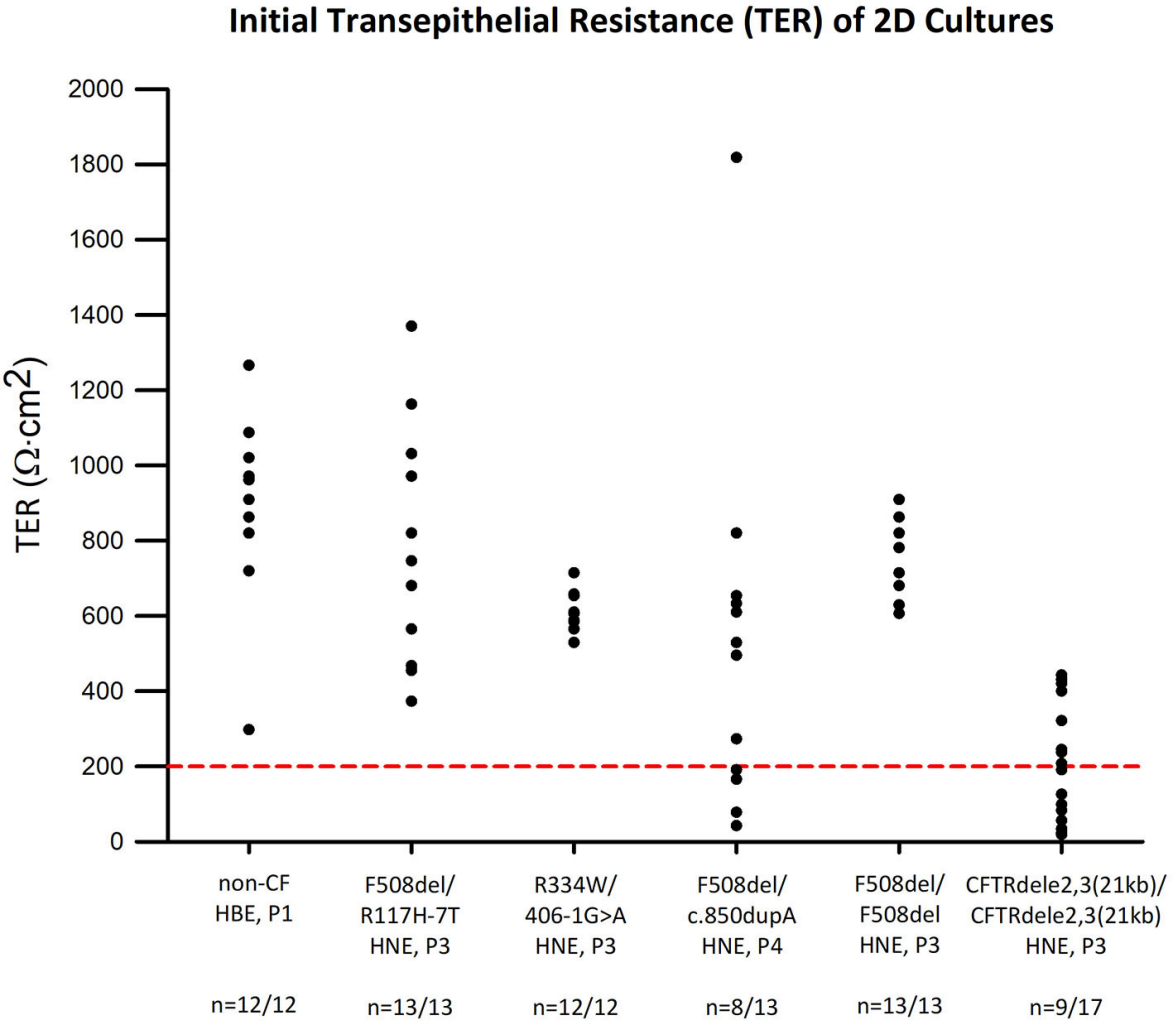
Transepithelial electrical resistance (TER) of CF and non-CF airway cultures. TER values were obtained at the beginning of the Ussing chamber experiment and plotted for each genotype. Red line represents quality control standard set at 200 Ωcm^2^. Except for F508del/c.850dupA cells (*n* = 8 of 13) and CFTRdele2,3(21 kb) homozygous cells (*n* = 9 of 17), all other cultures meet the standard and are viable for subsequent analysis.

All experiments were subject to an initial quality control cut-off of a minimum TER of 200 Ωcm^2^ before amiloride addition. This procedure allowed for selection against leaky cultures that perform poorly in Ussing chambers. All cultures from non-CF control (*n* = 12), F508del/R117H-7T (*n* = 13), R334W/406-1G>A (*n* = 12), and F508del/F508del (*n* = 13) cells passed. Eight out of 13 F508del/c.850dupA and 9 out of 17 CFTRdele2,3(21 kb) homozygous cultures passed the quality control criterion (Figure 3). Overall, 67 out of 80 experiments passed the quality control criterion for subsequent electrophysiologic analysis yielding a pooled success rate of 84%.

### CFTR Function and Modulator Responses of Non-CF Cultures

CFTR function and responsiveness to CFTR modulators were assessed after sodium currents were blocked by amiloride. CFTR currents in presence of forskolin across non-CF cultures (17.85 ± 2.21 μA/cm^2^) served as a reference standard for normal CFTR function and was set as 100%. The F508del/R117H-7T genotype served as an internal control standard to compare CFTR function measurement of the other CF genotypes to a mild CFTR function mutation.

**Figure 4:**
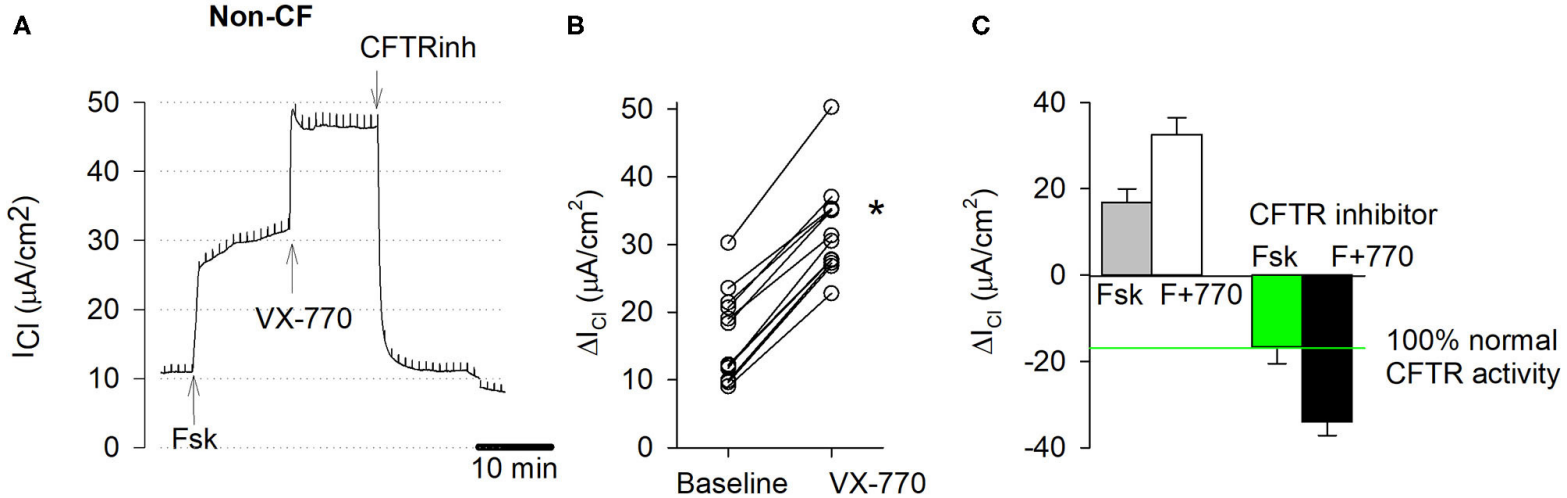
CFTR function measurements in EpiX^TM^-expanded non-CF cultures. Normal bronchial epithelial cells, one donor, passage 1. **(A)** Original short circuit current trace, untreated. Transepithelial chloride current (I_Cl_) was recorded in presence of amiloride. Stimulation of wildtype CFTR currents by the cAMP agonist forskolin (20 μM, serosal) and CFTR potentiator VX-770 (1 μM, mucosal) and subsequent inhibition by CFTR inhibitors (CFTRinh-172, PPQ-102, GlyH-101; 50 μM, mucosal). Note, trace does not show initial amiloride and final ATP additions. **(B)** Individual responses. Baseline represents change in cAMP-dependent CFTR current by forskolin alone. VX-770 represents change in current by forskolin+VX-770. * denotes significant increase by VX-770 (paired *t*-test, *p* < 0.05, *n* = 12). **(C)** CFTR-specific currents represent change in current due to stimulation by forskolin (cAMP) and VX-770 (upward bar) and total inhibition of cAMP-stimulated and cAMP+VX-770 stimulated CFTR currents by CFTR inhibitors (downward bar). Note that 100% normal CFTR activity is defined as the blocked forskolin-stimulated CFTR activity. Bars represent mean ± SE (*n* = 12).

Figure 4A shows a typical recording of an Ussing chamber experiment for the activation and block of CFTR-mediated chloride currents in non-CF cultures. Figure 4B shows individual responses to forskolin alone (baseline) and corresponding responses to acute stimulation by CFTR potentiator VX-770. Figure 4C summarizes chloride current changes in non-CF cultures. The forskolin and forskolin+VX-770-stimulated CFTR currents are plotted upward and corresponding blocked currents are plotted downward. Addition of forskolin alone stimulated CFTR currents by 16.51 ± 1.86 μA/cm^2^ (*n* = 12). The forskolin-stimulated CFTR current was further increased by 15.73 ± 0.70 μA/cm^2^ in response to the CFTR potentiator VX-770. Inhibition of the forskolin+VX-770 stimulated current by CFTR inhibitors yielded a blocked CFTR current (ΔI_CFTR_) of −34.42 ± 1.77 μA/cm^2^. The blocked forskolin-stimulated CFTR current averaged 17.85 ± 2.21 μA/cm^2^ (*n* = 12) and was used as a reference for the physiological CFTR activity and is referred to as baseline CFTR activity in this study. CFTR activities of bronchial vs. nasal non-CF cultures as well as ENaC and CaCC activities are provided in (Figure S7).

### CFTR Function and Modulator Responses of CF Cultures of Five CF Genotypes

**Figure 5:**
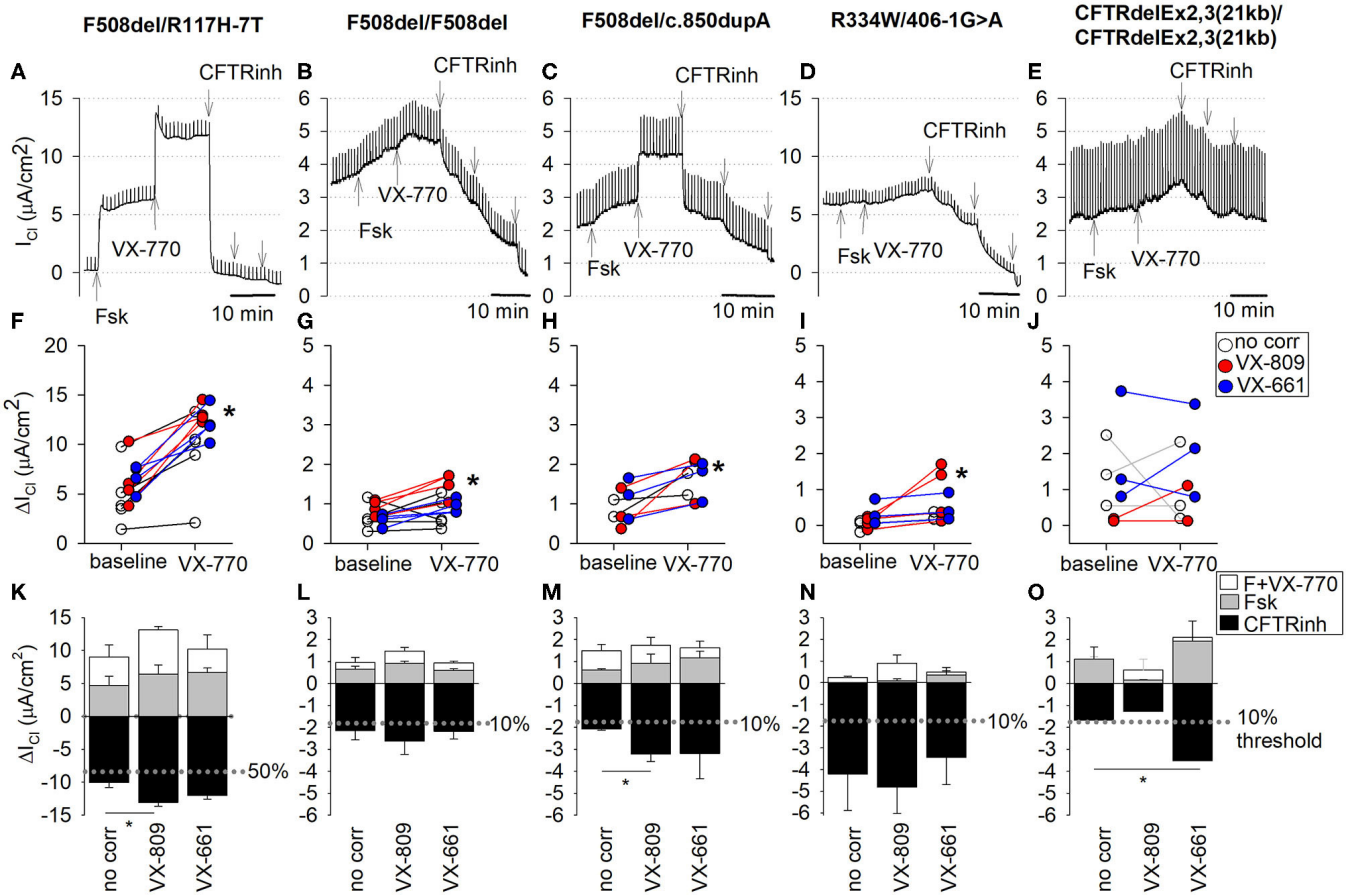
CFTR function measurements in EpiX^TM^-expanded CF patient cultures. CF nasal epithelial cultures, five CF subjects with different genotypes. **(A–E)** *Top*. Detailed recordings of CFTR currents. Traces were from cultures treated with CFTR corrector VX-809. Chloride currents (I_Cl_) were recorded in presence of amiloride. Note differences in scale, reflective of decreasing CFTR function with CF genotypes. CFTRinh refers to sequential addition of three CFTR inhibitor compounds (CFTRinh-172, PPQ-102, and GlyH-101). *Middle* **(F–J)**: Individual responses to forskolin (baseline) and forskolin plus VX-770 (VX-770). Baseline represents change in cAMP-dependent CFTR current by forskolin alone. VX-770 represents change in current by forskolin+VX-770. *denotes significant increase by VX-770 (paired *t*-test, *p* < 0.05). *Bottom* **(K–O)**: Average stimulated and blocked CFTR currents of non-treated cultures (no corr) and cultures treated with CFTR corrector VX-809 or VX-661. Upward bars present chloride current change (ΔI_Cl_) after stimulation with forskolin alone (cAMP; gray) or after stimulation with forskolin plus VX-770 (white, VX-770). Downward bars present ΔI_Cl_ after maximal block by CFTR inhibitors (CFTRinh; black). Bars represent mean ± SE. *, significantly different by unpaired *t*-test (*p* < 0.05). **(A,F,K)** F508del/R117H-7T, passage 3 (PD = 8.7). Non-treated: *n* = 5; VX-809 treated: *n* = 4; VX-661 treated: *n* = 4. **(B,G,L)** R334W/406-1G>A, passage 3 (PD = 8.7). Non-treated: *n* = 3; VX-809 treated: *n* = 4; VX-661 treated: *n* = 3. **(C,H,M)** F508del/c.850dupA, passage 4 (PD = 11.6). Non-treated: *n* = 2; VX-809 treated: *n* = 3; VX-661 treated: *n* = 3. **(D,I,N)** F508del homozygote, passage 3 (PD = 10.4). Non-treated: *n* = 5; VX-809 treated: *n* = 4; VX-661 treated: *n* = 4. **(E,J,O)** CFTRdele2,3(21 kb) homozygote, passage 3 (PD = 10.4). Non-treated: *n* = 4; VX-809 treated: *n* = 2; VX-661 treated: *n* = 3.

CFTR function and CFTR modulator responses were further tested in CF nasal cultures according to the aforementioned experimental protocol applied to non-treated and corrector-treated CF cultures (VX-809 or VX-661, overnight). CFTR current traces are shown for each genotype in Figures 5A–E. Individual responses to forskolin alone (baseline) and corresponding responses to CFTR potentiator VX-770 are shown in Figures 5F–J. Average chloride current changes elicited by forskolin, forskolin+VX-770, and CFTR inhibitors are summarized as bar charts for untreated, VX-809-treated, or VX-661-treated CF cultures (Figures 5K–O).

#### F508del/R117H-7T

All measured CFTR currents for all cultures in this study are summarized in Table 2. CF nasal cultures with the F508del/R117H-7T genotype were examined at passage 3 (PD = 8.7) and demonstrated robust stimulatory responses to both forskolin and VX-770 that were fully blocked by CFTR inhibitors (Figure 5A). The blocked forskolin-stimulated CFTR current, averaged 7.23 ± 1.20 μA/cm^2^ (*n* = 5) corresponding to 40% WT CFTR currents under baseline conditions (no CFTR potentiator, no CFTR corrector). Maximal activation of mutant CFTR currents in F508del/R117H-7T by forskolin+VX-770 restored ~56% of WT CFTR activity without prior CFTR corrector treatment (Figure 5K) and 73 and 67% after correction with VX809 and VX-661, respectively (Table 2). Overall, both CFTR correctors improved CFTR function of the F508del/R117H-7T genotype compared to non-treated cells.

**Table 2:** Summary of CFTR measurements in EpiX^TM^ expanded CF nasal cultures.

**Summary of CFTR Function and Modulator Responses** 100% WT CFTR = −17.85 ± 2.21 μA/cm^**2**^ (n=12)			
**Genotype**	**Treatment**	**CFTR current (μA/cm**^**2**^**, mean** **±** **SE)**	**Normal CFTR (%)**
F508del/R117H-7T	No corrector	−10.04 ± 0.69 (*n* = 5)	56
F508del/R117H-7T	VX-809	−13.06 ± 0.53 (*n* = 4)^*^	73
F508del/R117H-7T	VX-661	−12.01 ± 0.47 (*n* = 4)	67
F508del/F508del	No corrector	−2.14 ± 0.39 (*n* = 5)	12
F508del/F508del	VX-809	−2.63 ± 0.52 (*n* = 4)	15
F508del/F508del	VX-661	−2.19 ± 0.30 (*n* = 4)	12
F508del/c.850dupA	No corrector	−2.07 ± 0.04 (*n* = 2)	12
F508del/c.850dupA	VX-809	−3.21 ± 0.35 (*n* = 3)^*^	18
F508del/c.850dupA	VX-661	−3.19 ± 1.15 (*n* = 3)	18
R334W/406-1G>A	No corrector	−4.20 ± 1.38 (*n* = 3)	24
R334W/406-1G>A	VX-809	−4.81 ± 1.06 (*n* = 4)	27
R334W/406-1G>A	VX-661	−3.43 ± 1.03 (*n* = 3)	19
CFTRdele2,3(21 kb)/CFTRdele2,3(21 kb)	No corrector	−1.68 ± 0.32 (*n* = 4)	9
CFTRdele2,3(21 kb)/CFTRdele2,3(21 kb)	VX-809	−1.28 ± 0.05 (*n* = 2)	7
CFTRdele2,3(21 kb)/CFTRdele2,3(21 kb)	VX-661	−3.52 ± 0.05 (*n* = 3)^**^	20

All CFTR currents were measured in response to stimulation by forskolin and acute addition of VX-770 in uncorrected, VX-809 corrected or VX-661 corrected cultures. CFTR current refers to blockable current. Significance for CFTR corrector treatment is marked with

* (p < 0.05) and

** *(p < 0.01), determined by unpaired t-tests*.

#### F508del/F508del

CF nasal cultures with the F508del/F508del genotype were examined at passage 3 (PD = 10.4) and demonstrated small but detectable responses to forskolin that were slightly increased by VX-770 (Figure 5B, Table 2). Corrector treatment showed a tendency of increased current, however, statistical significance for treatment by VX-809 or VX-661 was not observed in our study (Figure 5L, Table 2).

#### F508del/c.850dupA

CF nasal cultures with the F508del/c.850dupA genotype were examined at passage 4 (PD = 11.6) and demonstrated a robust response to VX-770 and CFTR inhibitors blocked the stimulated currents (Figure 5C). In untreated cultures, currents averaged 12% of WT CFTR. Corrector-treated cultures restored up 18% of WT CFTR function and both VX-809 and VX-661 appeared equally effective (Figure 5M, Table 2).

#### R334W/406-1G>A

CF nasal cultures with the R334W/406-1G>A genotype were examined at passage 3 (PD = 8.7). Small currents were activated by forskolin and by VX-770 (Figure 5D). Uncorrected cultures showed CFTR currents of 24% of WT CFTR and currents were not significantly increased by correction (Figure 5N, Table 2).

#### CFTRdele2,3(21 kb)/CFTRdele2,3(21 kb)

CF nasal cultures of homozygous CFTRdele2,3(21 kb) genotype were examined at passage 3 (PD = 10.4) and demonstrated a small but detectable response to forskolin. A small increase by VX-770 was detected in three out of nine experiments. The stimulated currents were blocked by CFTR inhibitors suggesting some functional CFTR activity in this large deletion mutation (Figure 5E). Uncorrected cultures showed CFTR currents corresponding to 9% of WT CFTR. VX-661 correction appeared effective with an increase of current to 20% of WT CFTR, while VX-809 treatment showed insignificant effects (Figure 5O, Table 2). Because the observed effects were unexpected in this large deletion mutation, recordings of all conditions of these cultures are shown in detail in the (Figure S6).

### Corroboration of Assay Results With Sweat Chloride Measurements

**Figure 6:**
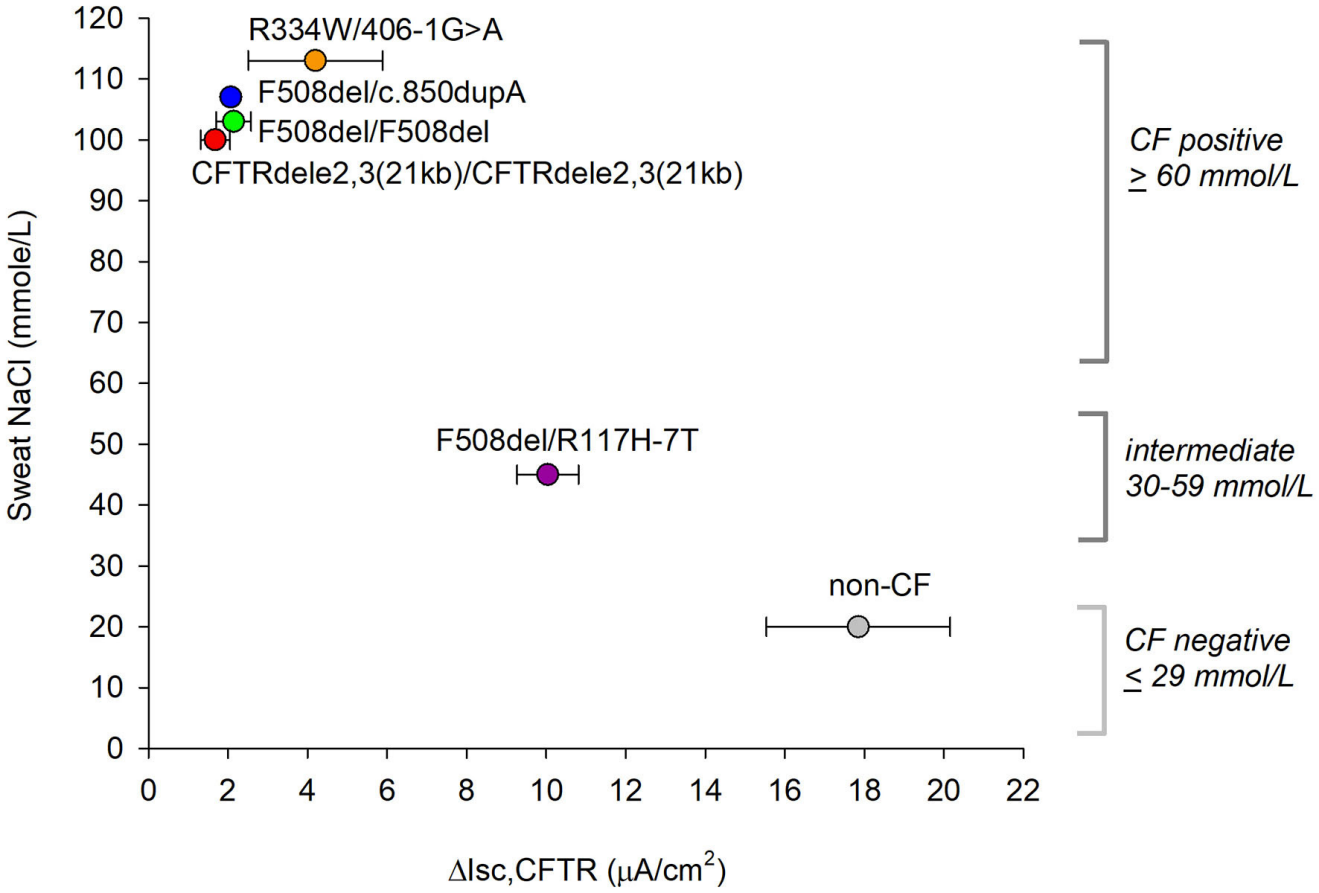
Relation between *ex vivo* CFTR current measurements and clinical sweat chloride concentrations. Official standards for CF diagnostics based on sweat chloride are shown on the right. ΔI_CFTR_ is presented as mean ± SE values of CFTR inhibitor-sensitive current recorded in forskolin plus VX-770 stimulated cells without CFTR corrector treatment. A relation between the two measures appears significant by Pearson correlation with *r* = −0.953, *p* = 0.003, normality passed (Shapiro-Wilk), *p* = 0.917.

To relate *in vitro* CFTR function data to a clinically relevant measure, we compared our results with *in vivo* sweat chloride measurements from individual patient charts. Sweat chloride was determined after stimulation of sweat using pilocarpine iontophoresis and collected using the Wescor Macroduct coils. The official standards for sweat chloride diagnostics from the CF Foundation are: (1) *Unlikely CF*: ≤ 29 mM, (2) *Intermediate:* 30 mM ≤ *x* ≤ 59 mM, (3) *Likely CF*: ≥60 mM (30). The CF individuals with genotypes CFTRdele2,3(21kb) homozygous, F508del homozygous, F508del/c.850dupA, and R334W/406-1G>A all recorded a sweat chloride concentration of >100 mM, indicating a severe CF phenotype. The F508del/R117H individual recorded a concentration of 45 mM, which was within an intermediate range (Figure 6). A correlation between *in vitro* CFTR function data (plotted as mean values of blocked CFTR currents obtained from non-treated nasal cultures) and clinical sweat chloride measurements was observed suggesting that the *in vitro* CFTR chloride current assay relates to the clinical sweat chloride measurement (*r* = −0.95, *p* = 0.003, Pearson correlation coefficient).

### Subject-Specific Differences in ENaC and CaCC Current Measurements

**Figure 7:**
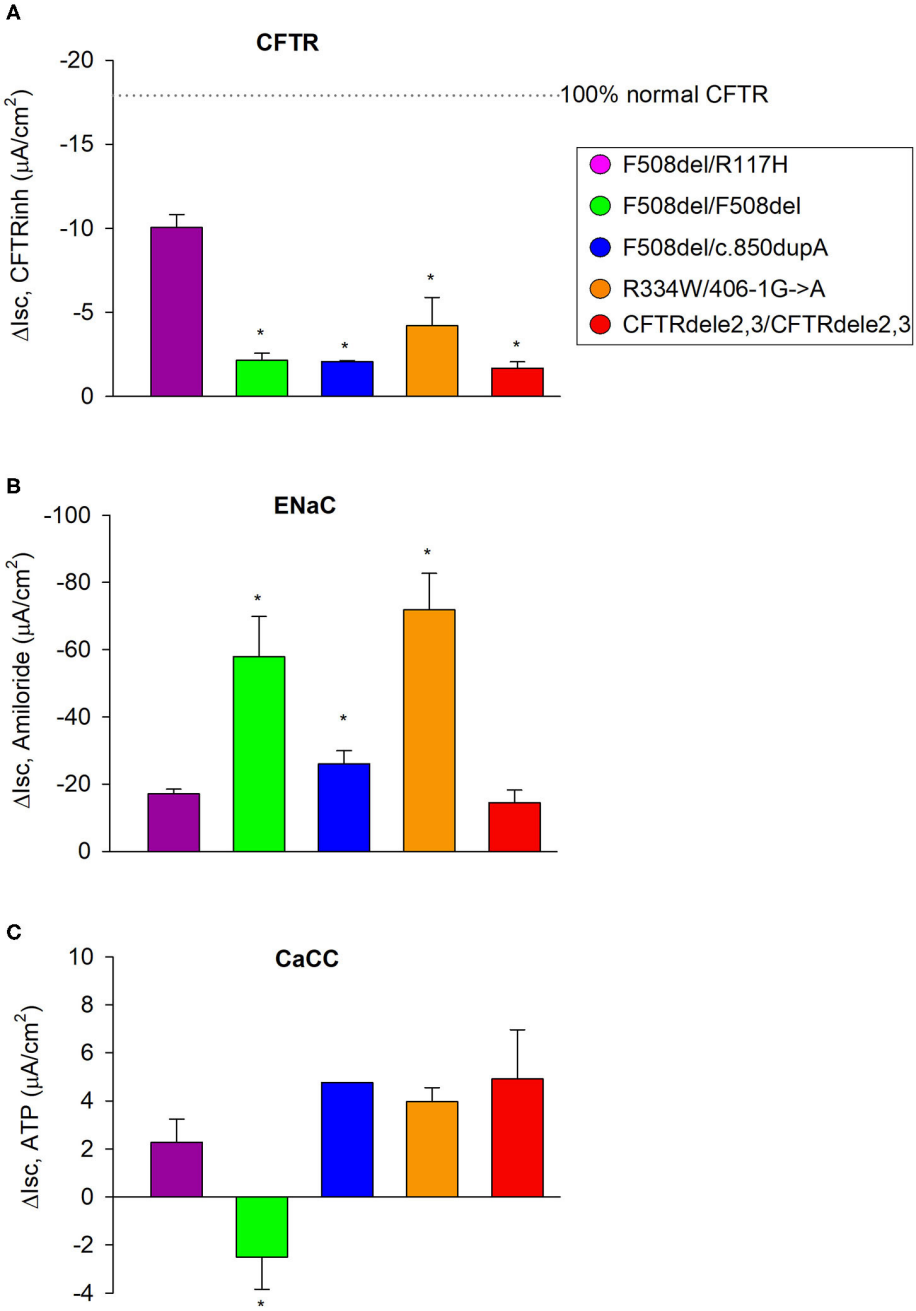
CFTR, ENaC, and CaCC activities of EpiX^TM^ expanded CF patient cultures. **(A)** The F508del/R117H-7H genotype was used as an internal reference to compare CFTR function among genotypes. CFTR function was quantified from the forskolin plus VX-770 stimulated current that was blockable by CFTR inhibitors and in cultures that received no CFTR corrector treatment. **(B)** Corresponding ENaC activity was quantified by measuring the change in current before and after addition of amiloride. Note that ENaC activity was significantly increased in cultures with the F508del/F508del or R334W/406-1G->A genotype. **(C)** Corresponding CaCC activity was quantified by measuring the change in current before addition of ATP and the maximal current stimulated by ATP (peak). Note that ATP induced a decrease in Isc in cultures with the F508del/F508del genotype suggesting that other conductances dominated the secretory response to ATP. All other cultures showed a chloride secretory response to ATP. Bars represent mean ± SE (*n* = 2–13 experiments per genotype). *denotes significantly different from F508del/R117H control group (Holm-Sidak, *p* < 0.05).

To control for the expression and activities of epithelial ion channels between the five CF subjects, we compared genotype-specific activities for CFTR, ENaC, and CaCC against cultures from the subject with the F508del/R117H genotype as a reference group (Figure 7A). ENaC and CaCC functions were tested by measuring the inhibition of Isc by amiloride (100 μM, to mucosa) at the beginning of the experiment and peak responses of Isc by ATP (500 μM, to mucosa) at the end of the experiment, respectively. Figure 7B summarizes ENaC currents among cultures from the five CF subjects. Isc decreased by −20.9 ± 1.6 μA/cm^2^ in F508del/R117H-7T cultures, −64.6 ± 4.8 μA/cm^2^ in F508del homozygous cultures, −21.3 ± 1.6 μA/cm^2^ in F508del/c.850dupA cultures, −75.8 ± 3.7 μA/cm^2^ in R334W/406-1G>A cultures, and −13.4 ± 2.6 μA/cm^2^ in CFTRdele2,3(21 kb) cultures. These data suggest that ENaC activity was significantly increased in cultures with the F508del/F508del genotype (3.1-fold) and R334W/406-1G->A genotype (3.6-fold). Prior treatment with VX-809 or VX-770 did not induce changes in ENaC activity. The effects of CFTR corrector treatment on ENaC and CaCC activities are summarized in Supplemental Materials for each genotype (Figures S2–S6).

ATP caused currents to increase transiently in all genotypes except in cultures with the F508del/F508del genotype where ATP caused a transient downward current (Figure 7C). ATP-induced CaCC activation increased currents that peaked at 2.2 ± 0.6 μA/cm^2^ in F508del/R117H-7T cells, −1.4 ± 0.7 μA/cm^2^ in F508del homozygous cells, 4.9 ± 1.0 μA/cm^2^ in F508del/c.850dupA cells, 4.2 ± 0.5 μA/cm^2^ in R334W/406-1G>A cells, and 5.2 ± 0.9 μA/cm^2^ in CFTRdele2,3(21kb) cells. Comparison against the F508del/R117H-7T control group suggest that CaCC activity is similar among cultures except for the subject with the F508del/F508del that showed an inverse peak response. Assessment of ENaC and CaCC activities in non-CF cultures are shown in Figure S7. Non-CF HBE cells expanded by EpiX^TM^ technology were characterized by an amiloride response of −3.3 ± 0.2 μA/cm^2^ and an ATP response of 8.4 ± 0.6 μA/cm^2^. ENaC activity was low in both bronchial and nasal non-CF cultures when compared to CF cultures. The discrepancy may be explained by variations in the ALI differentiation media and/or by subject-specific differences among non-CF cultures.

## Discussion

This study reports the utility of the EpiX^TM^ cell culture technology to expand airway epithelial cells from nasal brushing samples of CF patients for *in vitro* human cell-based models to test individual responses to CFTR-directed therapeutics. Zhang et al. (7) have previously shown that EpiX^TM^ medium supports over trillion-fold of *in vitro* expansion of bronchial epithelial cells under serum-free, feeder-free culture conditions. The expanded cells can be seamlessly integrated into exciting downstream differentiation protocols and put into electrophysiological CFTR function evaluations. The present work builds on this previous work and shows that the technology is applicable to culturing cells obtained from pediatric CF patients using nasal brushings as opposed to rectal or nasal biopsies or bronchoscopy but employing a much less invasive sampling method.

Nasal epithelial cells were collected by a simple nasal brushing procedure in children as young as 3 years and included three children between the age of 3–6 years and two adolescents of 17 and 20 years. The procurement of nasal cells was less invasive compared to an earlier study in adult CF patients (8) which required a topical anesthetic to harvest a sufficient amount of cells for further cell expansion by use of 3T3 feeder-cells and Rho-kinase inhibitor, a technology also known as conditional reprogramming (14). For the present study, we decided to explore the feeder-free EpiX^TM^ technology for nasal cell expansion. Our results showed that as few as 20,000 viable cells were sufficient to enable the proliferation and supply of 50 million patient cells within 1 month. This is a remarkable finding and demonstrates a reduction in starting material by a factor that is 50–100 times less when compared to the conditional reprogramming method. Our study extends the application of the EpiX^TM^ cell culture technology from bronchial epithelial cells to nasal epithelial cells. The derivation of adequate amounts of biomaterials from a small number of basal stem cells can be readily achieved by a simple nasal brushing procedure. The nominal number of viable cells that are required from the nasal brush sample allows for a very brief nasal brushing procedure that is well-tolerated in children without the need for local anesthetics. The EpiX^TM^-expanded patient cells were grown on cell culture inserts under air-liquid interface conditions and developed typical features of well-differentiated airway cell models including transepithelial resistance, amiloride-blockable sodium currents, CFTR and calcium-activated chloride currents.

The successful establishment of tight ALI cultures from EpiX^TM^-expanded cells is represented here by the metric of transepithelial resistance, a measure of epithelial barrier function (Figure 3). However, impairments in transepithelial resistances have been repeatedly reported for CF cultures including the F508del mutation that were associated with abnormalities in tight junction formation and attributed to the lack of CFTR expression (31). Here, we provide an instance of successfully differentiating nasal epithelial cells that harbor severe CF mutations into electrically tight ALI cultures which is a critical feature for Ussing chamber studies. We found that >80% of the EpiX^TM^-expanded CF nasal cells maintained epithelial integrity over 3 to 4 passages which is a motivating result. The need for epithelial barrier formation is a caveat for Ussing chamber studies and is in contrast to other fluorescence-based assays; however, short-circuit current measurements are superior in terms of sensitivity and ability to quantify vectorial transport of sodium and chloride ions across epithelial layers.

Our characterization of mutant CFTR function both supports and extends the standard literature on these CF variants. R117H-7T, for instance, is already established as a CF mutation with mild phenotypes when inherited with the longer 7T polymorphism (23, 24), which is supported in our characterization of an intermediate CFTR function. CFTR modulator responses for the R117H/F508del genotype are in a similar range reported by Gentzsch et al. (16).

The mild channel conductance mutation, R334W (25), is similarly characterized here as a functional variant that conserves 24% wildtype function, even when in combination with the 406-1G>A haplotype which is a severe splice mutation (26). In comparison, the two frameshift and non-sense mutations, c.850dupA and CFTRdele2,3(21 kb), are expected to show little to no CFTR function because of the anticipated absence of complete protein synthesis and processing. Our findings from CFTRdele2,3(21 kb) homozygous cultures, however, suggest the presence of a small but detectable residual CFTR on the plasma membrane, since current changes were detected upon acute exposure to CFTR-specific small molecules.

CFTRdele2,3(21 kb) is a large deletion mutation and known to cause severe CF. It was initially classified as a Class I mutation and was recently presented as an example for a class 7 mutation (no mRNA) (32), although it was shown originally that mRNA transcripts of CFTRdele2,3 were similar to wildtype (22). Here, we found that nasal epithelial cell cultures homozygous for the CFTRdele2,3(21 kb) mutation responded to VX-770 and to corrector VX-661 in the Ussing assay, which was unexpected (Figure 5O). Based on the disease genomics, we initially hypothesized that homozygous CFTRdele2,3(21 kb) will result in no functional CFTR protein in the plasma membrane. A cluster of acidic residues (at positions 47, 51, 54, and 58) in the amino-terminal cytoplasmic tail is deleted and therefore not available to control CFTR ion channel gating through an intramolecular interaction with the regulatory R-domain (33). However, it is not known to what degree the deleted regulatory cluster will impair the correction of CFTRdele2,3(21 kb) activity. Given that CFTRdele2,3(21 kb) produces a stop codon as early as codon 106 (22), unconventional explanations, such as the use of alternative stop codons (34, 35), may provide further insight into the mechanism of disease of CFTRdele2,3(21 kb). Through this functional characterization of CFTRdele2,3(21 kb), we provided a framework for a better understanding of the disease mechanism of CFTRdele2,3(21 kb), as the cells' response to CFTR-specific compounds suggested some localization of functional CFTR in the apical cell membrane. Alternative explanations for CFTR synthesis in CFTRdele2,3(21 kb), such as the use of downstream start codons, may therefore find support through our findings (34, 35).

This is the first electrophysiological study that examined CFTR activity in ALI cultures from a homozygous CFTRdele2,3(21 kb) patient with the Ussing assay. However, our experimental approach was limited by the inherent low transepithelial resistance of the CFTRdele2,3(21 kb) cultures and the few cultures that developed TER values >200 Ω.cm^2^ (9 out of 17). The current tracings and side-by-side comparison of non-treated, VX-809- and VX-661 treated CFTRdele2,3(21 kb) cultures (Figures S6F–H) illustrate a small but detectable improvement of CFTRinh-blocked currents and in particular after correction with VX-661. However, based on the limitations of this study it will be important to perform future studies with a larger number of ALI cultures and to include additional assays that are not limited by low TER values (e.g., single cell patch clamp recordings, iodide influx, forskolin-induced swelling assays, immune-fluorescent staining of CFTR).

Because VX-661 and VX-770 restored a fraction of CFTR function of CFTRdele2,3(21kb), there is a possibility that clinical benefits may be reached. The newly FDA-approved triple combination, Trikafta® – an Elexacaftor (VX-445), Tezacaftor (VX-661) and Ivacaftor (VX-770) combination – is a promising candidate for CFTRdele2,3(21 kb) based on our results we obtained for the dual VX-661 and VX-770 combination. Further studies are warranted that are aimed at determining if VX-445 will also improve function for the homozygous CFTRdele2,3(21 kb) genotype. Moving forward, EpiX^TM^ expanded nasal cells can optimize results even further by (1) testing new approved treatment options (Trikafta®) as well as next generation and investigational compounds [e.g., WX_corr_-A23, WX_corr_-B09 (36)], and (2) by including protein or mRNA level analyses to expand from an electrophysiological characterization to an underlying disease mechanisms of each variant. Specifically, analyzing Trikafta® responsiveness would be most important and relevant for future assays, given the drug's suitability and standard of care for the F508del variant (37).

After completion of the study, we noted the following limitations that affected the strength of our data: First, the EpiX^TM^ -expanded non-CF cultures were of bronchial origin while the CF cultures were of nasal origin. This was a result of the limitation of procuring nasal cells from healthy children, which did not pass the risk-to-benefit determination during study planning. Second, some ALI cultures, and in particular CFTRdele2,3(21 kb) had lower resistances than others, which can affect proper voltage clamping (owing to an unfavorable ratio of the epithelial and the fluid resistance) and consequently the short-circuit current measurement; however, the magnitude of the gradient-driven chloride currents was low in amiloride-treated cultures indicating that Isc measurements were performed on CFTRdele2,3(21 kb) cultures that were not leaky (Figure S6A). Third, during the course of this study, VX-661 and VX-809 were the only available correctors; the newly approved corrector VX-445 might have provided additional insights and needs to be tested in a future study. Forth, the number of ALI cultures was limited to 2–3 experimental runs per group and a larger study would be needed to establish patient-specific and genotype-specific CFTR functionality and differences in CFTR modulator responses.

Clinically, our study provides an example of bed-to-bench cooperation that can help optimize treatment for all patients with cystic fibrosis. Patients with rare CFTR variants have previously been underrepresented in many large CF pharmacology trials and many have not been eligible for CFTR modulator therapy (38, 39). The development of patient-derived cell models for electrophysiologic CFTR function tests may ultimately provide a theranostic tool and help to decrease health disparities in this patient population. In light of recent advancements in personalized CF therapy (17), such models of institutional co-operation in healthcare research exemplify the feasibility of personalized medicine, especially for diseases such as CF. The improvement of *ex vivo* assays by creating patient-derived culture models can provide a theranostic tool to support the pursuit of personalized medicine and a more efficient relationship between the patient, clinician and the scientist at large.

## Data Availability Statement

All datasets presented in this study are included in the article/Supplementary Material.

## Ethics Statement

The studies involving human participants were reviewed and approved by IRB#2018-048; Institutional Review Board, UCSF Benioff Children's Hospital Oakland. Written informed consent to participate in this study was provided by the participants' legal guardian/next of kin.

## Author Contributions

BI, JP, and AS performed experiments. NL and EG performed cell procurement. JP and BI wrote the manuscript with critical input from CZ, BP, NL, and WF. All authors contributed to the article and approved the submitted version.

## Conflict of Interest

AS and BP are employees of Propagenix Inc.; BP is an equity holder in Propagenix Inc. The remaining authors declare that the research was conducted in the absence of any commercial or financial relationships that could be construed as a potential conflict of interest.
