# Supplementary figures and images for: Functional Profiling of CFTR-Directed Therapeutics Using Pediatric Patient-Derived Nasal Epithelial Cell Models
Source: Front Pediatr. 2020 Sep 4;8:536. doi: 10.3389/fped.2020.00536 (PMC7500161; doi:10.3389/fped.2020.00536)

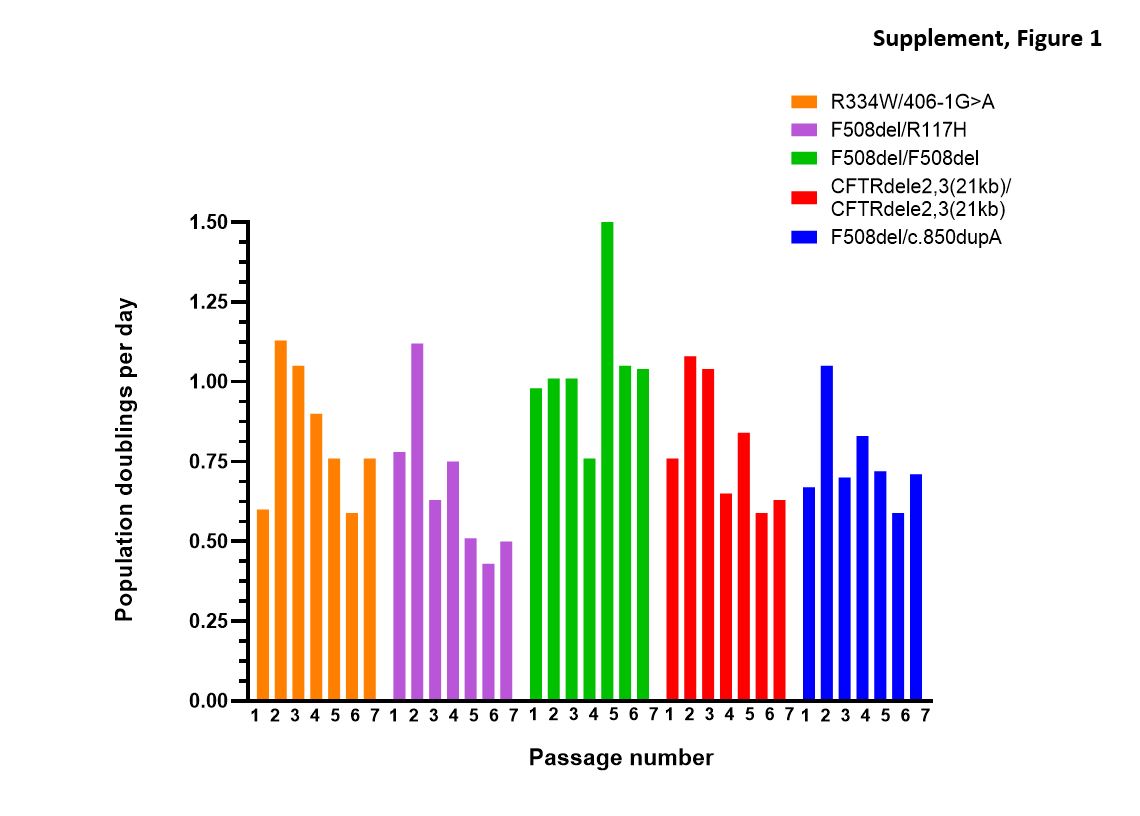

Supplement: Supplementary file 1 [file Image_1.jpg]

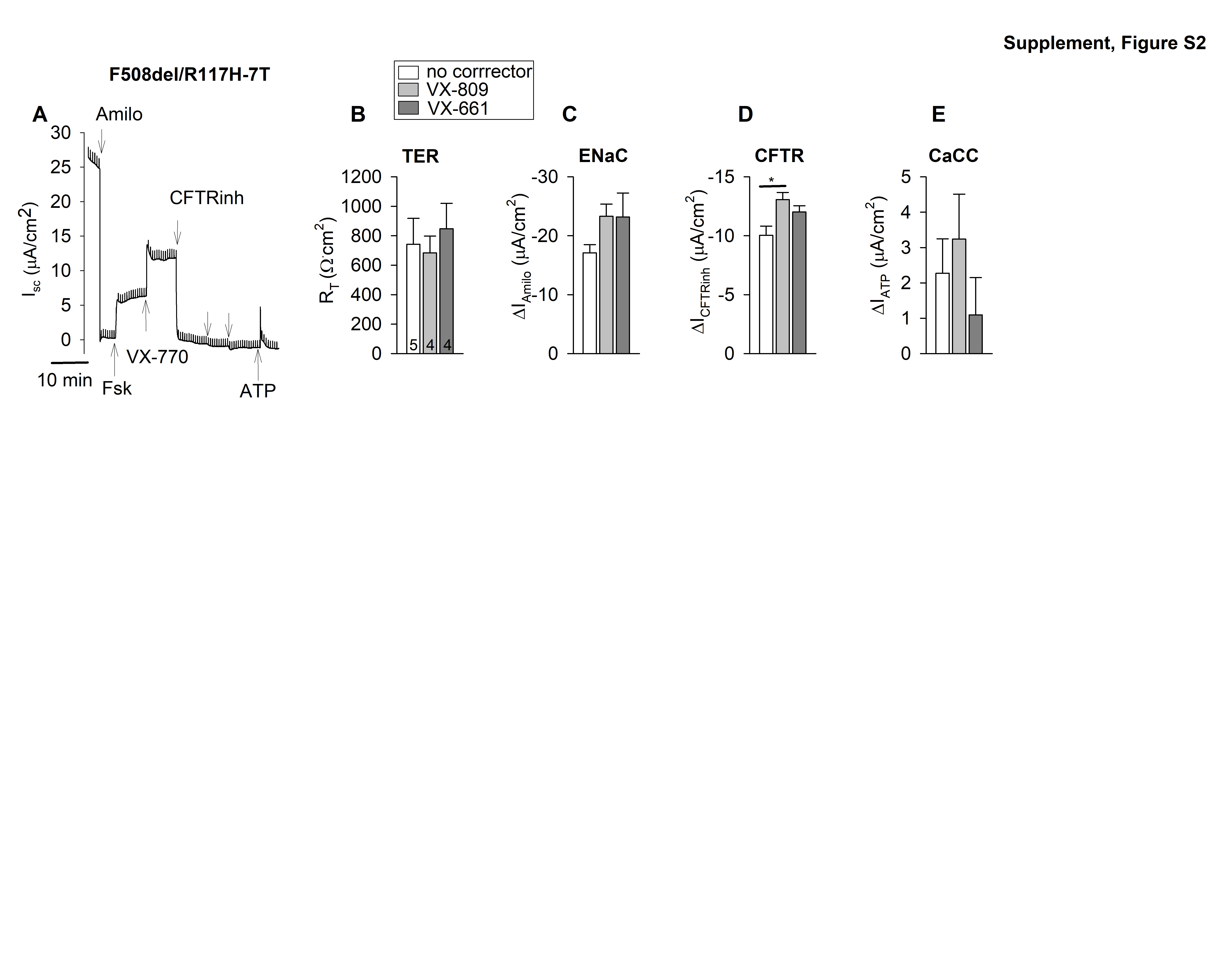

Supplement: Supplementary file 2 [file Image_2.jpg]

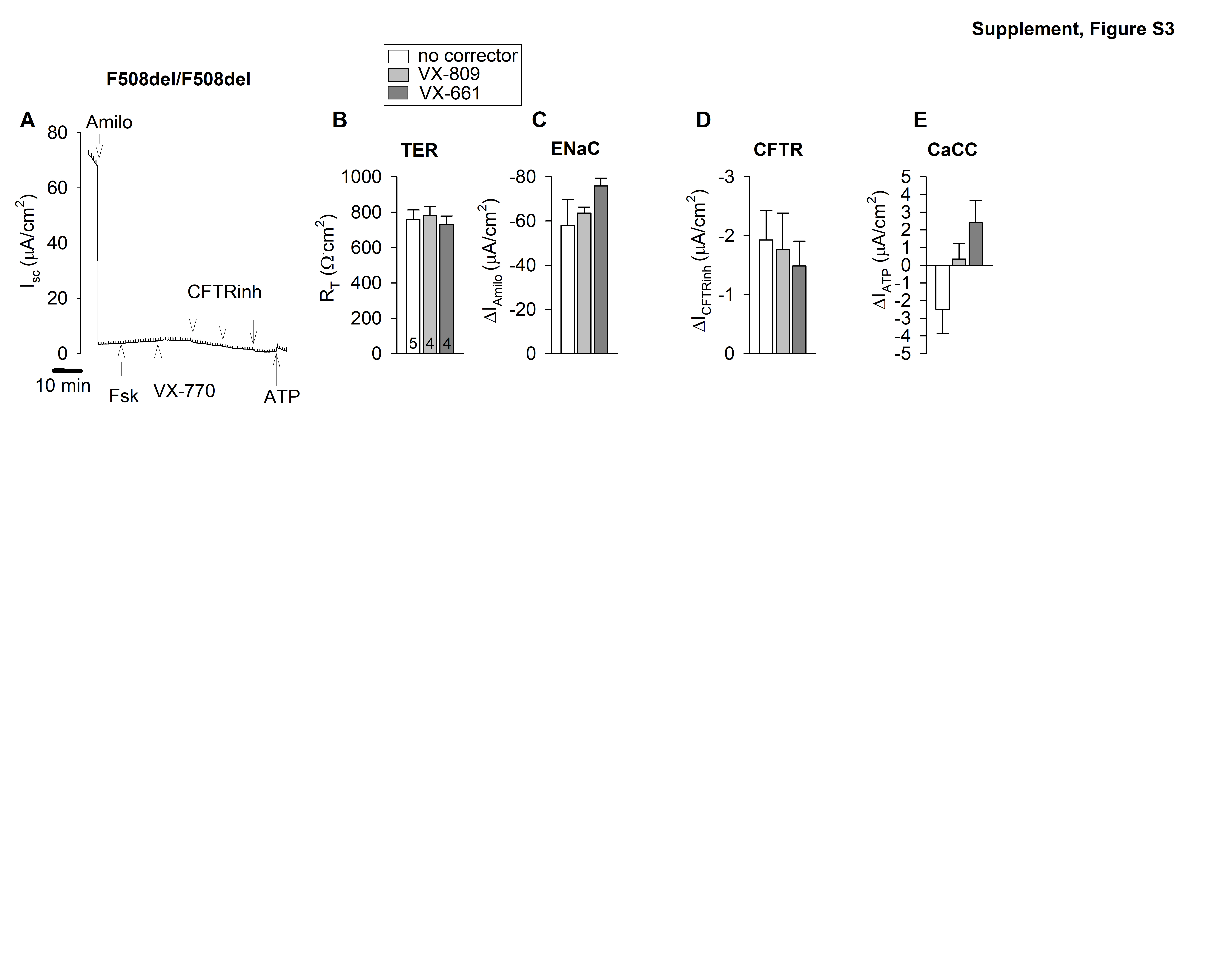

Supplement: Supplementary file 3 [file Image_3.jpg]

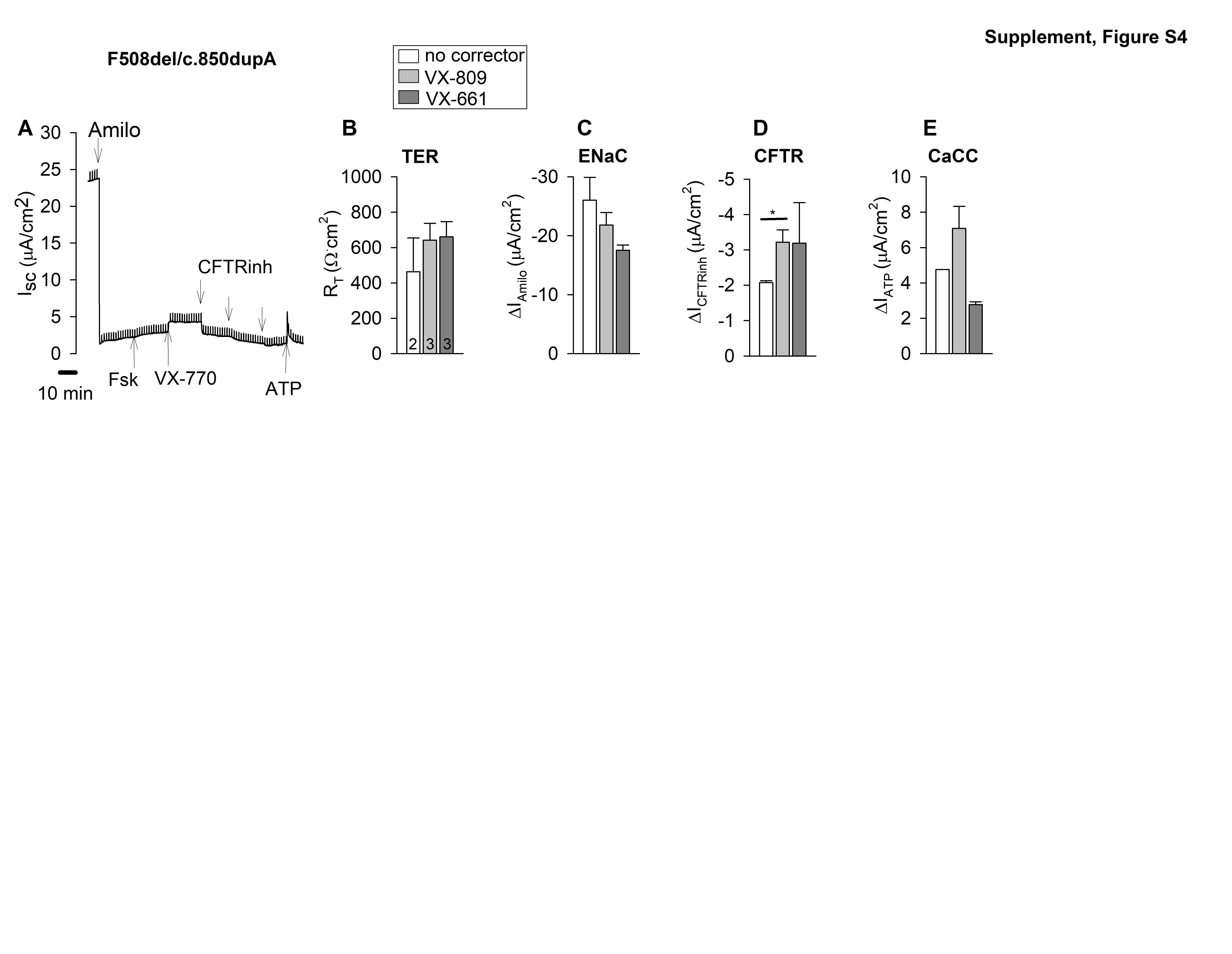

Supplement: Supplementary file 4 [file Image_4.jpg]

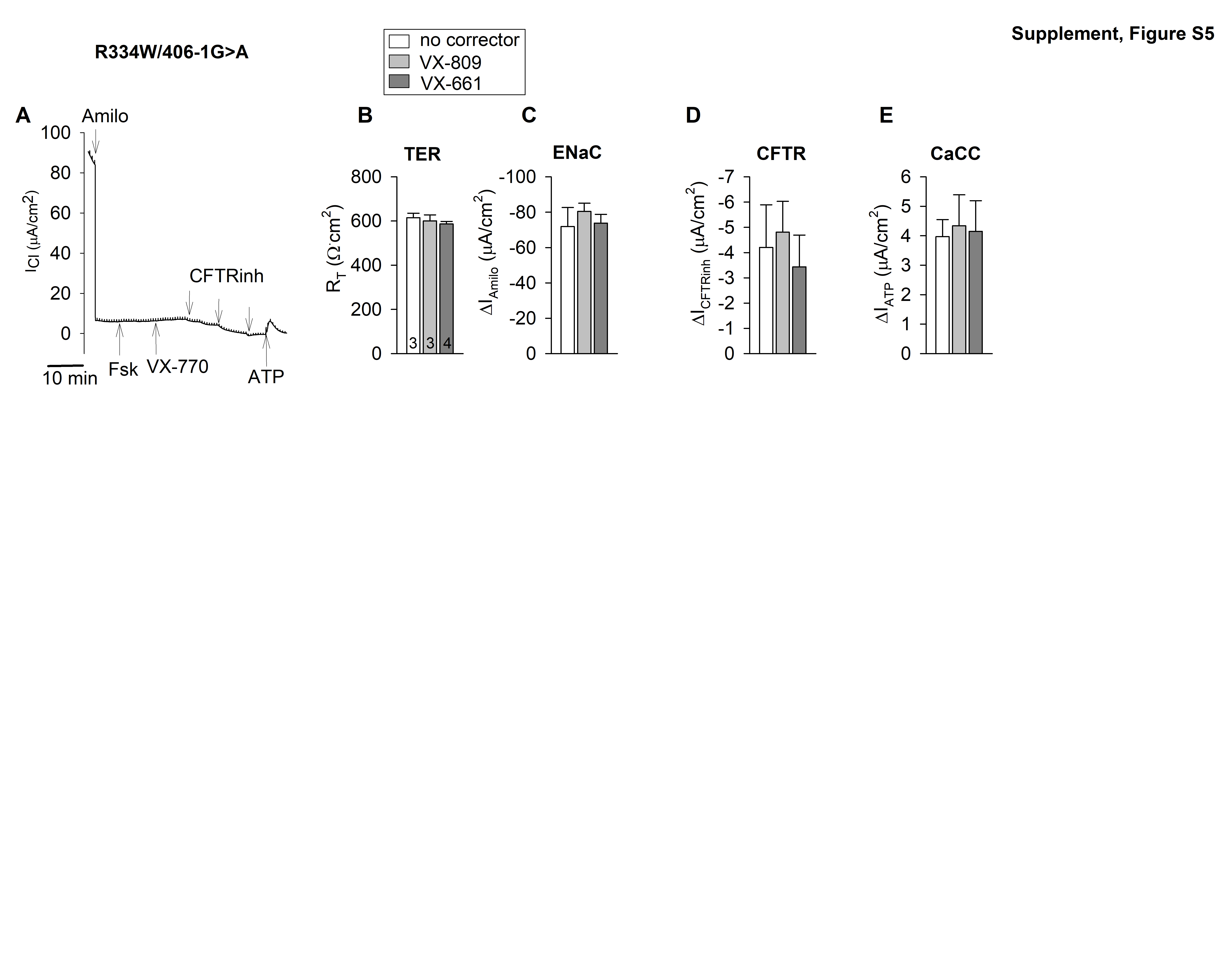

Supplement: Supplementary file 5 [file Image_5.jpg]

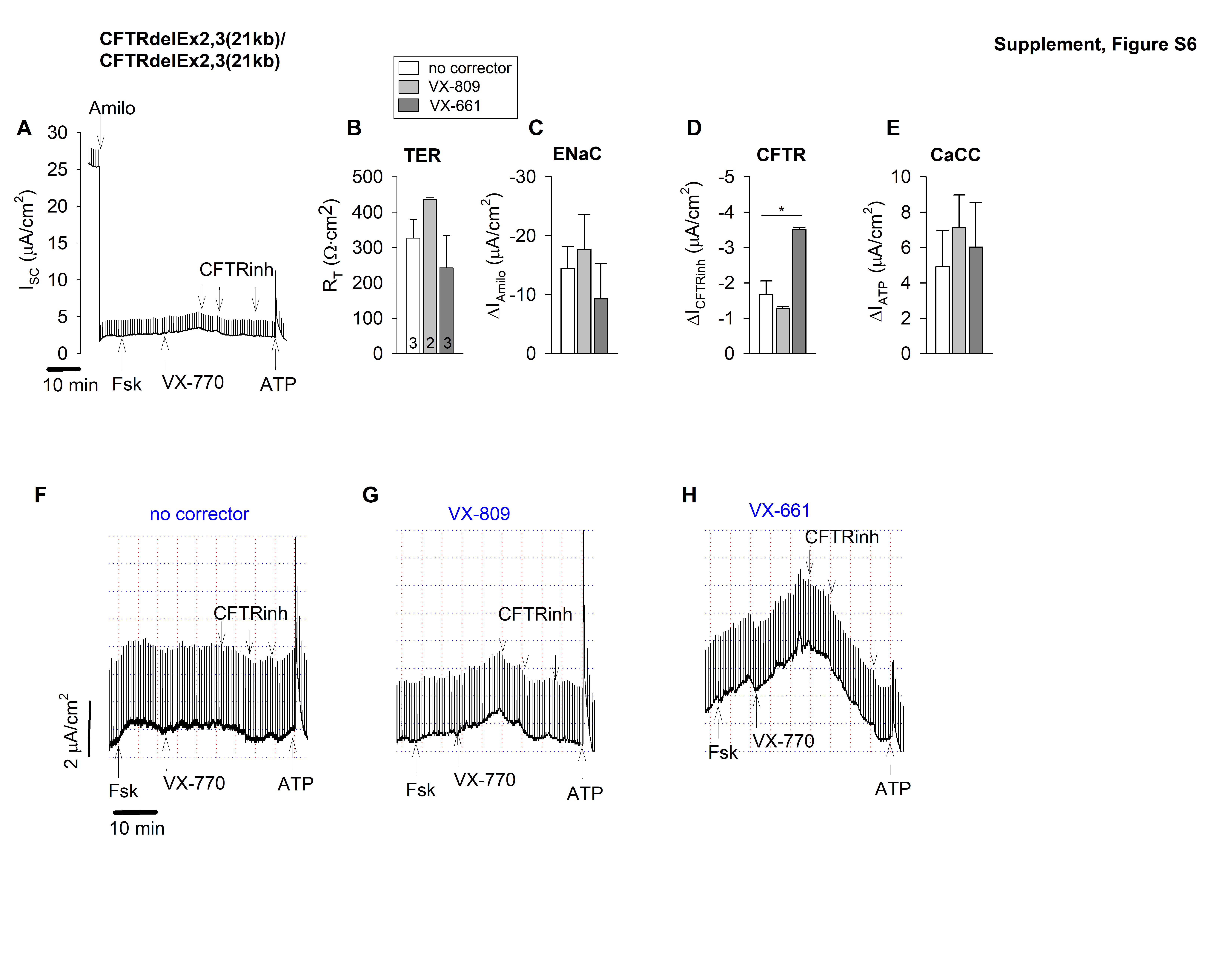

Supplement: Supplementary file 6 [file Image_6.jpg]

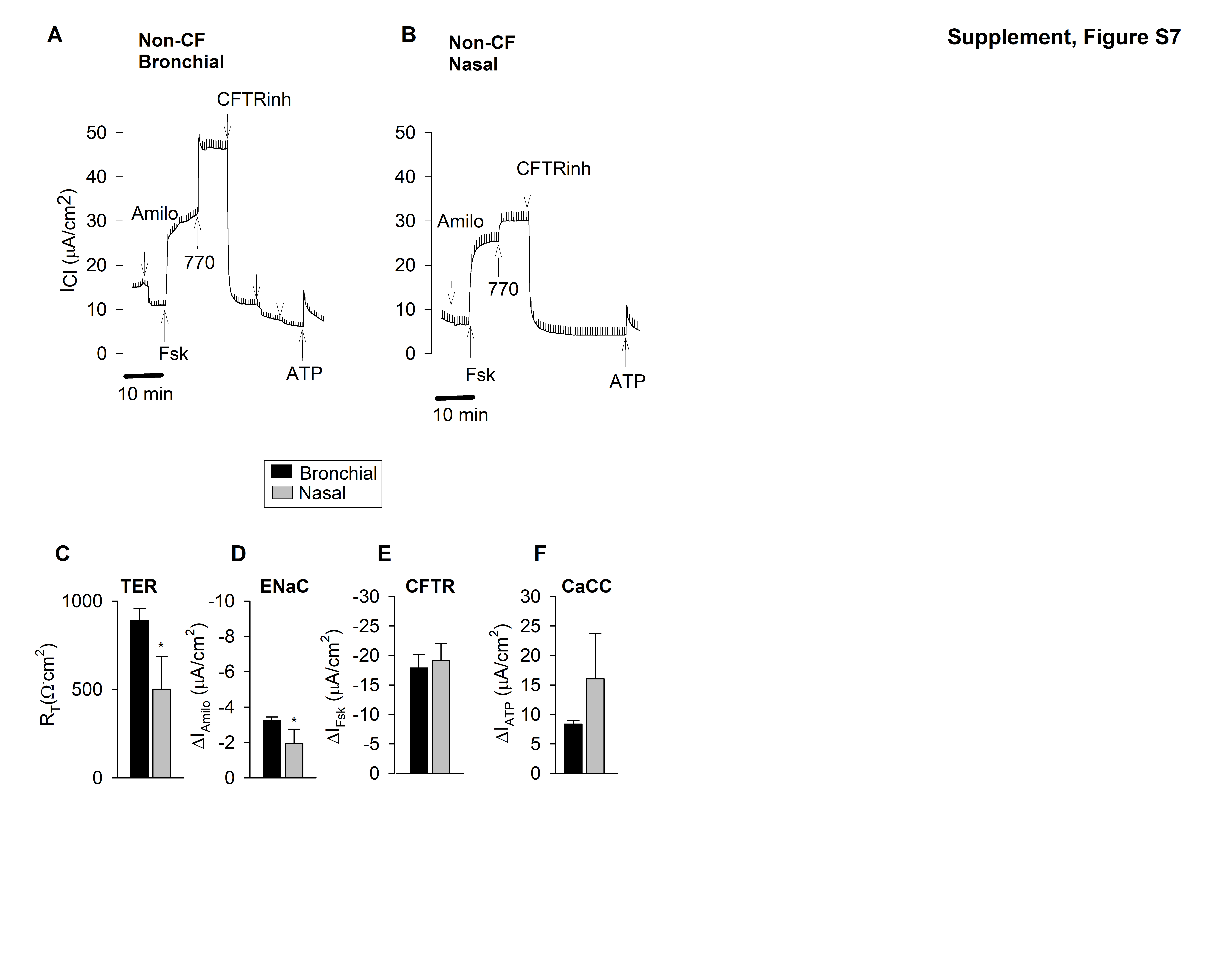

Supplement: Supplementary file 7 [file Image_7.jpg]
