# Supplementary material for: Functional Profiling of CFTR-Directed Therapeutics Using Pediatric Patient-Derived Nasal Epithelial Cell Models
Source: Front Pediatr. 2020 Sep 4;8:536. doi: 10.3389/fped.2020.00536 (PMC7500161; doi:10.3389/fped.2020.00536)
Supplement: Supplementary file 8 [file Data_Sheet_1.pdf]

Supplementary Material

1 Supplementary Figures and Tables

Table S1. Final concentrations of components in collection medium for nasal brushings.

Component	Final Concentration
LHC Basal Medium<br>(ThermoFisher Scientific, Cat. #12-677-019)	Base Media
Penicillin Streptomycin (Penicillin: 10,000U/mL, Streptomycin: 10,000 μg/mL)<br>(ThermoFisher Scientific, Cat. #15140-122)	200 μg/mL
Gentamicin (50mg/mL)<br>(ThermoFisher Scientific, Cat. #15750-060)	20 μg/mL
Amphotericin B (250μg/mL)<br>(ThermoFisher Scientific, Cat. #15290-018)	2.5 μg/mL

Table S2. Final concentrations of media components in the Vertex ALI Differentiation Medium (V-ALI).

Component	Final Concentration
Base Media	100% DMEM/F12
Ultroser G	2%
Fetal Clone II	2%
Bovine Brain Extract	0.0225 mg/mL
Triiodothyronine (T3)	0.5 μM
Transferrin	0.03125 μM
Ethanolamine	0.25 μM
Phosphorylethanolamine	0.25 μM
Hydrocortisone	0.02 μM
Epinephrine	1.5 μM
Insulin	0.435 μM
Retinoic Acid	0.01 μM
Penicillin Streptomycin	100 μg/mL

Figure S1. Expansion of CF nasal cells in EpiX^{TM} medium. Population doubling per day in CF cultures from five donors. Isolated primary human nasal cells from nasal brushings were grown in EpiX^{TM} medium. Population doublings were maintained between 0.5-1 per day at passages 1, 2, 3, 4, 5, 6, and 7.

Figure S2. Personalized ion transport measurement of EpiX™-expanded CF nasal cultures from a 6-year-old CF patient with F508del/R117H-7T genotype. 2D-Planar nasal cultures were generated from EpiX™-propagated cells (P3/8.7PD). **A.** Overview recording**.** Short circuit current trace (I_{SC}, µA/cm^2) recorded in Ussing chamber in presence of a serosal to mucosal Cl gradient. I_{sc} was measured in response to amiloride (Amilo; 100 µM, apical), followed by forskolin (Fsk; 10 µM, serosal) and VX-770 (1 µM, apical). To reassure complete block of CFTR activity for accurate quantification of mutant CFTR activity, three different CFTR inhibitors were added sequentially (black arrows; CFTRinh172: 50-100 µM; apical. PPQ102: 50-100 µM, apical; GlyH101: 50 µM, apical). CaCC activity was measured in response to ATP (500 µM, apical). To visualize transepithelial resistance (TER), a marker of epithelial barrier function, upward current deflections (caused by 1 mV voltage pulses, applied for a duration of 1 s and every 60s) are included in the original recording. The recording shown was obtained from a VX-809 treated tissue at ALI day 26 and was selected as a representative trace of n=13 experiments. B-D. Quantification of transepithelial electrical resistance (TER), ENaC, CFTR and CaCC activities of non-treated (n=5) cultures (ctr) and cultures treated overnight with CFTR corrector VX-809 (n=4) or VX-661 (n=4). **B.** Transepithelial resistance values (R_T, in Ω·cm^2) were taken before amiloride was added and were similar among groups **C.** Isc changes in response to amiloride (ΔI_{Amilo}) were similar among groups. **D.** CFTR activity was estimated from the maximal inhibition of the forskolin plus VX-770-stimulated Isc by CFTR inhibitors. CFTR activity was increased in the VX-809 treated group. **E.** ATP-induced chloride secretory responses (ΔI_{ATP}) were measured at peak values and were similar among groups. Data are presented as mean values±SE. *denotes significantly different from control (P<0.05, Holm-Sidak)

Figure S3. Personalized ion transport measurement of EpiX™-expanded CF nasal cultures from a 17-year-old CF patient with F508del/F508del genotype. 2D-Planar nasal cultures were generated from EpiX™-propagated cells (P3/10.4PD). **A.** Overview recording. Short circuit current trace (I_{SC}, µA/cm^2) recorded in Ussing chamber in presence of a serosal to mucosal Cl gradient. I_{sc} was measured in response to amiloride (Amilo; 100 µM, apical), followed by forskolin (Fsk; 10 µM, serosal) and VX-770 (1 µM, apical). To reassure complete block of CFTR activity for accurate quantification of mutant CFTR activity, three different CFTR inhibitors were added sequentially (black arrows; CFTRinh172: 50-100 µM; apical. PPQ102: 50-100 µM, apical; GlyH101: 50 µM, apical). CaCC activity was measured in response to ATP (500 µM, apical). To visualize transepithelial resistance (TER), a marker of epithelial barrier function, upward current deflections (caused by 1 mV voltage pulses, applied for a duration of 1 s and every 60s) are included in the original recording. The recording shown was obtained from a non-treated culture at ALI day 25 and was selected as a representative trace of n=13 experiments. B-D. Quantification of transepithelial electrical resistance (TER), ENaC and CaCC activities of non-treated (n=5) cultures (ctr) and cultures treated overnight with CFTR corrector VX-809 (n=4) or VX-661 (n=4). **B.** Transepithelial resistance values (R_T, in Ω·cm^2) were taken before amiloride was added. **C.** Current changes in response to amiloride (ΔI_{Amilo}) were similar among groups. **D.** CFTR activity was estimated from the maximal inhibition of the forskolin plus VX-770-stimulated Isc by CFTR inhibitors. Average CFTR activity

was not significantly different among groups. **E.** ATP-induced chloride secretory responses (ΔI_{ATP}) were measured at peak values and were similar among group. Data are presented as mean values±SE.

Figure S4. Personalized ion transport measurement of EpiX™-expanded CF nasal cultures from a 20-year-old CF patient with F508del/c.850dupA genotype. 2D-Planar nasal cultures were generated from EpiX™-propagated cells (P4/11.4PD). **A.** Overview recording. Short circuit current trace (I_{SC}, µA/cm^2) recorded in Ussing chamber in presence of a serosal to mucosal Cl gradient. I_{sc} was measured in response to amiloride (Amilo; 100 µM, apical), followed by forskolin (Fsk; 10 µM, serosal) and VX-770 (1 µM, apical). To reassure complete block of CFTR activity for accurate quantification of mutant CFTR activity, three different CFTR inhibitors were added sequentially (black arrows; CFTRinh172: 50-100 µM; apical. PPQ102: 50-100 µM, apical; GlyH101: 50 µM, apical). CaCC activity was measured in response to ATP (500 µM, apical). To visualize transepithelial resistance (TER), a marker of epithelial barrier function, upward current deflections (caused by 1 mV voltage pulses, applied for a duration of 1 s and every 60s) are included in the original recording. The recording shown was obtained from a VX-809 treated culture at ALI day 27 and was selected as a representative trace of n=8 experiments. B-D. Quantification of transepithelial electrical resistance (TER), ENaC and CaCC activities of non-treated (n=2) cultures (ctr) and cultures treated overnight with CFTR corrector VX-809 (n=3) or VX-661 (n=3). **B.** Transepithelial resistance values (R_T, in $\Omega \cdot cm^2$) were taken before amiloride was added and were similar among groups. **C.** Current changes in response to amiloride (ΔI_{Amilo}) were similar among groups. **D.** CFTR activity was estimated from the maximal inhibition of the forskolin plus VX-770-stimulated Isc by CFTR inhibitors. Average CFTR activity was increased in the VX-809 treated group. **E.** ATP-induced chloride secretory responses (ΔI_{ATP}) were measured at peak values and were similar among groups. Data are presented as mean values±SE. *denotes significantly different from control ($P<0.05$, unpaired t-test)

Figure S5. Personalized ion transport measurement of EpiX™-expanded CF nasal cultures from a 3-year-old CF patient with R334W/406-1G->A genotype. 2D-Planar nasal cultures were generated from EpiX™-propagated cells (P3/8.7PD). **A.** Overview recording. Short circuit current trace (I_{SC}, µA/cm^2) recorded in Ussing chamber in presence of a serosal to mucosal Cl gradient. I_{sc} was measured in response to amiloride (Amilo; 100 µM, apical), followed by forskolin (Fsk; 10 µM, serosal) and VX-770 (1 µM, apical). To reassure complete block of CFTR activity for accurate quantification of mutant CFTR activity, three different CFTR inhibitors were added sequentially (black arrows; CFTRinh172: 50-100 µM; apical. PPQ102: 50-100 µM, apical; GlyH101: 50 µM, apical). CaCC activity was measured in response to ATP (500 µM, apical). To visualize transepithelial resistance (TER), a marker of epithelial barrier function, upward current deflections (caused by 1 mV voltage pulses, applied for a duration of 1 s and every 60s) are included in the original recording. The recording shown was obtained from a VX-809 treated culture at ALI day 26 and was selected as a representative trace of n=10 experiments. B-D. Quantification of transepithelial electrical resistance (TER), ENaC and CaCC activities of non-treated (n=3) cultures (ctr) and cultures treated overnight with CFTR corrector VX-809 (n=4) or VX-661 (n=3). **B.** Transepithelial resistance values (R_T, in $\Omega \cdot cm^2$) were taken before amiloride was added and were similar among groups. **C.** Current changes in response to amiloride (ΔI_{Amilo}) were large and similar among groups. **D.** CFTR activity was estimated from the maximal inhibition of the forskolin plus VX-770-stimulated Isc by CFTR inhibitors. Average CFTR activity was not significantly different among groups. **E.** ATP-induced chloride secretory responses (ΔI_{ATP}) were measured at peak values and were similar among groups. Data are presented as mean values±SE.

Figure S6. Personalized ion transport measurement of EpiX™-expanded CF nasal cultures from a 4-year-old CF patient with CFTRdele2,3(21kb)/CFTRdele2,3(21kb) genotype. 2D-Planar nasal cultures were generated from EpiX™-propagated cells (P3/10.4PD). **A.** Overview recording. Short circuit current trace (I_{SC}, µA/cm^2) recorded in Ussing chamber in presence of a serosal to mucosal Cl gradient. I_{sc} was measured in response to amiloride (Amilo; 100 µM, apical), followed by forskolin (Fsk; 10 µM, serosal) and VX-770 (1 µM, apical). To reassure complete block of CFTR activity for accurate quantification of mutant CFTR activity, three different CFTR inhibitors were added sequentially (black arrows; CFTRinh172: 50-100 µM; apical. PPQ102: 50-100 µM, apical; GlyH101: 50 µM, apical). CaCC activity was measured in response to ATP (500 µM, apical). The recording was obtained from a VX-809 treated culture at ALI day 22 and was selected as a representative trace of n=9 experiments. B-D. Quantification of transepithelial electrical resistance (TER), ENaC and CaCC activities of non-treated (n=4) cultures (ctr) and cultures treated overnight with CFTR corrector VX-809 (n=2) or VX-661 (n=3). **B.** Transepithelial resistance values (R_T, in Ω·cm^2) were taken before amiloride was added and were similar among groups **C.** I_{sc} changes in response to amiloride (ΔI_{Amilo}) were similar among groups. **D.** CFTR-mediated chloride currents ($\Delta I_{CFTRinh}$) were determined by calculating maximal inhibition of the forskolin plus VX-770-stimulated Isc by CFTR inhibitors. CFTR currents were increased in the VX-661 treated group. **E.** ATP-induced chloride secretory responses (ΔI_{ATP}) were measured at peak values and were similar among groups. Data are presented as mean values±SE. *denotes significantly different from control (P<0.05, Holm-Sidak). **F, G, H.** Detailed recordings of CFTR currents to illustrate CFTR modulator effects on homozygous CFTRdele2,3 mutation (ALI cultures, day 22). Original traces recorded in presence of amiloride. Axes are scaled the same for all three plots. **F.** Untreated, **G.** VX-809-treated, **H.** VX-661-treated. CFTRinh-blocked currents were -0.67 µA/cm^2 (nontreated), -1.34 µA/cm^2 (VX-809), and -3.54 -0.67 µA/cm^2 (VX-661) suggesting rescue of CFTRdele2,3(21kb) by CFTR corrector compounds. Note, size of current deflections indicate that TER values were in a similar range; R_T=322 Ω·cm^2 (no corrector), R_T=431 Ω·cm^2 (VX-809), R_T = 400 Ω·cm^2 (VX-661).

Figure S7. Personalized ion transport measurements of non-CF cultures. 2D-Planar cultures were generated from **A.** EpiX™-propagated bronchial epithelial cells (passage 1) from an adult non-CF donor with idiopathic pulmonary fibrosis and **B.** nasal epithelial cells (passage 1) propagated by conditional reprogramming. Expanded cells were differentiated by a similar ALI protocol as previously described (8). A,B. Overview recording. Short circuit current trace (I_{SC}, µA/cm^2) recorded in Ussing chamber in presence of a serosal to mucosal Cl gradient. I_{sc} was measured in response to amiloride (Amilo; 100 µM, apical), followed by forskolin (Fsk; 10 µM, serosal) and VX-770 (1 µM, apical). To reassure complete block of CFTR activity for accurate quantification of mutant CFTR activity, three different CFTR inhibitors were added sequentially (black arrows; CFTRinh172: 50-100 µM; apical. PPQ102: 50-100 µM, apical; GlyH101: 50 µM, apical) in recording A, whereas a single CFTR inhibitor (CFTRinh172) was applied in recording B. CaCC activity was measured in response to ATP (500 µM, apical). To visualize transepithelial resistance (TER), a marker of epithelial barrier function, upward current deflections (caused by 1 mV voltage pulses, applied for a duration of 1 s and every 60s) are included in the original recording. C-F. Quantification of transepithelial electrical resistance (TER), CFTR, ENaC and CaCC activities of bronchial and nasal cultures **C.** Transepithelial resistance values (R_T, in Ω·cm^2) were taken before amiloride was added and were lower in the nasal cultures **D.** Current changes in response to amiloride (ΔI_{Amilo}) are small and decreased in the nasal culture. **E.** Block of forskolin-stimulated CFTR current by CFTR inhibitors (ΔI_{Fsk}) are similar among bronchial and nasal cultures **F.** ATP-induced chloride secretory responses (ΔI_{ATP}) were plotted from peak values and were similar among groups. Data are presented

as mean values±SE (n=3-12 experiments). *denotes significantly different between groups ($P \leq 0.05$, t-test).
